# The Effects of Graded Levels of Calorie Restriction: XIV. Global Metabolomics Screen Reveals Brown Adipose Tissue Changes in Amino Acids, Catecholamines, and Antioxidants After Short-Term Restriction in C57BL/6 Mice

**DOI:** 10.1093/gerona/glz023

**Published:** 2019-04-09

**Authors:** Cara L Green, Sharon E Mitchell, Davina Derous, Yingchun Wang, Luonan Chen, Jing-Dong J Han, Daniel E L Promislow, David Lusseau, Alex Douglas, John R Speakman

**Affiliations:** 1 School of Biological Sciences, Institute of Biological and Environmental Sciences, University of Aberdeen, Scotland, UK; 2 State Key Laboratory of Molecular Developmental Biology, Institute of Genetics and Developmental Biology, Chinese Academy of Sciences, China; 3 Key Laboratory of Systems Biology, Innovation Center for Cell Signaling Network, Institute of Biochemistry and Cell Biology, China; 4 Chinese Academy of Sciences Key Laboratory of Computational Biology, Chinese Academy of Sciences-Max Planck Partner Institute for Computational Biology, Shanghai Institutes for Biological Sciences, Chinese Academy of Sciences, China; 5 Department of Pathology and Department of Biology, University of Washington at Seattle

**Keywords:** Thermogenesis, Diabetes, Dopamine, Torpor, Obesity

## Abstract

Animals undergoing calorie restriction (CR) often lower their body temperature to conserve energy. Brown adipose tissue (BAT) is stimulated through norepinephrine when rapid heat production is needed, as it is highly metabolically active due to the uncoupling of the electron transport chain from ATP synthesis. To better understand how BAT metabolism changes with CR, we used metabolomics to identify 883 metabolites that were significantly differentially expressed in the BAT of C57BL/6 mice, fed graded CR (10%, 20%, 30%, and 40% CR relative to their individual baseline intake), compared with mice fed ad libitum (AL) for 12 hours a day. Pathway analysis revealed that graded CR had an impact on the TCA cycle and fatty acid degradation. In addition, an increase in nucleic acids and catecholamine pathways was seen with graded CR in the BAT metabolome. We saw increases in antioxidants with CR, suggesting a beneficial effect of mitochondrial uncoupling. Importantly, the instigator of BAT activation, norepinephrine, was increased with CR, whereas its precursors l-tyrosine and dopamine were decreased, indicating a shift of metabolites through the activation pathway. Several of these key changes were correlated with food anticipatory activity and body temperature, indicating BAT activation may be driven by responses to hunger.

Brown adipose tissue (BAT) is found in many mammals (1,2). The principal role of BAT is thermogenesis (3), which is activated when organisms require additional heat to raise body temperature, such as during arousal from torpor, or hibernation (4–6). BAT activity is regulated through the sympathetic nervous system by the release of norepinephrine, and during activation, large amounts of both glucose and lipids are consumed to facilitate heat production via mitochondrial uncoupling protein 1 (UCP1) (3). Because of this process, BAT has become very interesting in terms of aging-associated diseases such as type 2 diabetes and obesity (7,8).

Several studies have indicated that manipulation of BAT can result in increased metabolism and weight loss. Stimulation of BAT using a β3 adrenoreceptor agonist in obese Sprague–Dawley rats increased resting metabolic rate (by 40%–45%), increased UCP1 content in BAT, reduced abdominal white adipose tissue (WAT), reversed diet-induced obesity and caused browning of WAT despite no change in food intake (9). Chronic infusion of secretin—a non-sympathetic BAT activator—in obese mice can increase energy expenditure (10). Surgical transplantation of BAT similarly elevates metabolic rate and heat production causing fat and weight loss in mice (11–13) and reversal of type 1 diabetes (14). Increasing UCP1 activity has also been shown to protect against obesity in aP2-Ucp transgenic genetically obese agouti viable yellow mice and reduce subcutaneous fat in aP2-Ucp C57BL/6J mice (15). Recently, it has been shown that BAT activation can induce satiation in humans through the gut hormone secretin, making it an attractive target for weight loss interventions (10).

Up until now, the most robust nonpharmacological intervention for reducing obesity and type 2 diabetes is calorie restriction (CR), which is also associated with reducing age-associated disorders including neurodegeneration and cancer (16,17). Not only does CR improve health parameters with age, but it also increases life span, although this is yet to be established in humans (18,19). The benefits of CR have been seen across many organisms from nematodes to primates (19,20). In rodents, CR has been shown to have a linear effect on life span: as calorie intake decreases, life span increases, up to at least 65% restriction (21,22). Whether BAT has a significant role in CR-mediated improvements in glycemic control and weight loss has yet to be seen.

CR leads to browning of WAT into functional BAT-like beige fat in lean male C57BL/6 and BALB/c mice after only 1 week of restriction (23). This may contribute to metabolic improvements with CR, including improved insulin sensitivity and glucose tolerance. However, studies using albino male mice on 50% CR for 3 weeks showed that the rate of BAT oxygen consumption actually decreased. But, the relative weight and protein content of BAT did not change and DNA content in the BAT increased by 93% indicating acceleration of cell proliferation (24).

It may be beneficial for mammals under CR to initiate browning, as during CR, rodents and primates decrease their core body temperature (25–27) and once body mass falls to a critically low level, they initiate periods of torpor, presumably in an effort to achieve energy balance (28). To acutely increase body temperature for periods of high activity during feeding (food anticipatory activity [FAA] (29)), it might be necessary to activate BAT.

In our previous work on the same individual mice used in this study, we have shown that, although BAT mass decreased with increased CR, this decrease was relatively small compared with other fat depots (30). It is also possible that during CR, BAT switches function to store lipids; proteomic analysis indicated that 9-month-old rats subject to 6 months of CR showed either a decline or no change in the mitochondrial electron transport chain but enhanced fatty acid biosynthesis (31). In addition, after 106 weeks of 40% CR, both *Ins1*^*+/−*^:*Ins2*^*−/−*^ and *Ins1*^*+/+*^:*Ins2*^*−/−*^ mice showed an increase in mass and whitening of the BAT, indicating an increase in the fat storage capacity of BAT with CR (32).

The discrepancy in changes of BAT mass with CR may be a result of several factors including amount of CR, length of restriction, and study species. In particular, though studies in rats and mice are often treated as comparable, rats are a nonhibernating species and do not enter torpor under restriction, and non-shivering thermogenesis is far more pronounced in mice. In addition, as mice are smaller, they produce more heat per unit mass. This may result in an inability for mice to increase BAT mass, which is energetically expensive (33–35).

As far as we are aware, no study to date has investigated the effects of CR on the BAT metabolome; however, lipid metabolism, amino acids, nucleotide pathways, and redox regulation have been shown to change in BAT after cold exposure in C57BL/6 mice (36). In particular, acute cold exposure increased serine levels, which has been observed in previous studies (37). Serine metabolic pathways are thought to affect glycolysis (38) and glutathione through mammalian target of rapamycin complex 1 (mTORC1) activity, which also has implications for cell proliferation and aging (39,40). Glutamine, which also accumulates in BAT during cold exposure, serves many metabolic purposes, including involvement in the TCA cycle through conversion to glutamic acid or α-ketoglutarate. It is a nitrogen donor that is needed for purine biosynthesis, this supports data that suggest DNA levels in BAT increase with CR (36).

Here, we characterize the metabolome of BAT in male C57BL/6 mice exposed to CR. We applied five levels of CR, 0%, 10%, 20%, 30%, and 40% relative to individually measured baseline intakes, for 3 months, expecting a graded change of the metabolome. Owing to the significant roles that BAT and UCP1 play in energy expenditure and regulation of adiposity, we explored how BAT metabolites changed with increasing CR.

## Methods

### Experimental Design

Our data set consisted of metabolomic data from BAT samples taken from 48 individuals across six different feeding groups, including four CR groups and two ad libitum (AL) fed groups. Full details of the experiment are available at the Open Science Framework (DOI: 10.17605/OSF.IO/9YATH) and in ref. (30). Briefly, male mice were randomly allocated to one of six treatment groups: 12AL (*n* = 8), 24AL (*n* = 9), 10% CR (*n* = 8), 20% CR (*n* = 7), 30% CR (*n* = 7), and 40% CR (*n* = 9). All animals were fed a high carbohydrate open source diet (D12450B: Research diets, NJ) that contains 20% protein, 70% carbohydrate, and 10% fat (by energy). Amount of restriction was determined based on food intake of individual mice over a 2-week baseline period. The 12AL group was used as the control instead of 24AL to remove the “time since last meal” effect as all mice had been food deprived for at least 7.5 hours before culling. We initiated CR at 20 weeks to avoid any effect of CR on development while retaining effectiveness of increasing life span (41). CR mice were fed at lights out (1830 hours) Mice were fed CR (or AL) diets for 12 weeks, before being sacrificed at 32 weeks of age. Mice were culled between 1400 and 1800 hours, BAT was snap frozen in liquid nitrogen before being stored at –80°C.

### Animals

Mice were purchased from Charles River (Ormiston, UK). All procedures were reviewed and approved by the University of Aberdeen Welfare and Ethical Review Board and carried out under a Home Office issued license compliant with the Animals (Scientific Procedures) Act 1986. The C57BL/6 strain is already known to live longer under CR (42,43). More information on additional procedures and measures conducted on these mice can be found in the first article of this series (30).

### BAT Metabolite Extraction

Individual frozen mouse BAT samples (≈ 25 mg) were homogenized using an ULTRA-TURRAX dispenser T-25 Basic (IKA, Staufen, Germany) at level 5 in 1,000 µL of chloroform:methanol:water (1:3:1) at 4°C. Samples were agitated for 1 hour at 4°C and centrifuged at 13,000*g* for 3 minutes at 4°C. Supernatant was aliquoted into 180 µL samples and stored under argon at –80°C.

### Liquid Chromatography–Mass Spectrometry

We used hydrophilic interaction liquid chromatography to detect metabolites in both positive and negative ionization modes. This was carried out on a Dionex UltiMate 3000 RSLC system (Thermo Fisher Scientific, Hemel Hempstead, UK) using a ZIC-pHILIC column (150 mm × 4.6 mm, 5 μm column, Merck Sequant). The column was maintained at 30°C and samples were eluted with a linear gradient of 20 mM ammonium carbonate in water (increasing from 20% to 95%) and acetonitrile (decreasing from 80% to 5%) more than 26 minutes at a flow rate of 0.3 mL/min. The injection volume was 10 μL and samples were maintained at 4°C before injection. For the mass spectrometry (MS) analysis, a Thermo Orbitrap Exactive (Thermo Fisher Scientific) was operated in polarity switching mode.

### MS Data Processing

To process, extract, and visualize peaks from raw data mzXML files, we used the package *xcms 1.52.0* (44) in the R statistical environment, version 3.4.0 (45). We extracted mass-to-charge ratios (*m*/*z*), retention times, and intensities for each sample for 2,754 peaks in the positive ionization mode and 3,155 in the negative ionization mode. We then used the package *MSCombine 1.1* (46) to combine data from the positive and negative ionizations modes, which allows us to filter metabolites identified in both ionization modes.

### Metabolite Identification

From the combined data set, we identified metabolites from the HMDB, KEGG, and LipidMaps databases using the package *xMSannotator 1.3.1* (47). Unknown metabolites were identified using a clustering algorithm that uses *m*/*z* values, retention times, intensities, and potential adducts and *xMSannotator* provides a confidence score. Metabolites with a confidence score of less than 2 were excluded from further analysis. In addition, metabolites with multiple matches were filtered based on the difference between their theoretical and actual monoisotopic mass and the metabolite ID with the smallest difference was kept, and others were removed.

### Metabolomic Preprocessing

As metabolites can exhibit significant stochastic variation it is necessary to filter out “noisy,” uninformative metabolites. Before metabolomic analysis, a series of processing steps were applied to the entire data set, to supply us with the most instructive and stable metabolites for further analysis. First, metabolites were normalized using a log base 2 transformation. Second, only metabolites with a signal-to-noise ratio (signal-to-noise ratio = mean/sample standard deviation) greater than or equal to 15 (48) were kept for analysis. Third, metabolites that were missing from 15% or more of all samples were removed. To fill in missing values from the remaining samples, we imputed using a random forest regression approach (49,50) using the missForest package (51) for metabolites that had peaks that were significant in at least one group.

### Statistical Modeling of Differential Metabolite Expression

To detect significantly differentially expressed metabolites between treatment groups, an empirical Bayes moderated linear model was fitted to each metabolite (52). The empirical Bayes approach shrinks the estimated sample variances by borrowing information from across metabolites. Comparisons across metabolite fold changes were made between each level of CR (10%, 20%, 30%, and 40%) and 24AL relative to 12AL. *p* Values for each comparison were adjusted using the Benjamini–Hochberg procedure using a false discovery rate (FDR) of 20% (53,54). We used the package Devium for the Orthogonal Partial Least Square Discriminate Analysis (O-PLS-DA) analysis to complete the validation steps and retrieve metabolite loadings (55). O-PLS-DA allows us to discriminate between groups in multivariate data and to determine the most influential metabolites (56). We compared our model fit to random chance by generating 1,000 permuted models, and by splitting the data into a pseudo-training/test set to simulate root mean square error of prediction. Test/training was randomly assigned to the 46 samples in a 12:34 ratio, 1,000 times.

### Biological Pathway Analysis

Pathway analysis was conducted in *mummichog* as part of the metabolite identification process and using Ingenuity Pathway Analysis (IPA). For analysis in *mummichog*, metabolites with an unadjusted *p* value of less than or equal to .05 generated by the empirical Bayes linear model for each comparison with 12AL were used, along with coefficients and retention times. *p* value cutoff was relaxed to allow for greater potential to detect relevant biological pathways. In addition, the IPA program allowed us to take advantage of the Ingenuity Knowledge Base, a repository of biological and chemical information from the literature. We entered unadjusted *p* values and coefficients from each comparison alongside IDs from either KEGG or HMDB.

### Correlations With Physiological Parameters

We tested for correlations between several physiological variables, including physical activity, FAA (29), body temperature and incidence of torpor (28), and normalized metabolite intensities for each individual mouse using Pearson correlation coefficients. Associated *p* values for each correlation were adjusted using the Benjamini–Hochberg procedure with a FDR of 5%.

## Results

### Characterization of BAT MS Profile

CR was applied to male mice at 20 weeks of age for 12 weeks. Two control groups were allowed ad libitum access to food for 12 hours (12AL) and 24 hours a day (24AL), and the four CR groups were given 10%, 20%, 30%, and 40% restriction from their baseline intakes (10CR, 20CR, 30CR, and 40CR). 12AL was used as the control group for all further analyses to remove any potential “time since last meal effects.” Using liquid chromatography–mass spectrometry we found 3,155 *m*/*z* features detected in the negative ionization mode and 2,754 *m*/*z* features in the positive ionization mode. Features were combined, which reduced the number of total features to 5,860 *m*/*z* features. After filtering (see Methodology section), there were 2,876 *m*/*z* features present. Missing values were imputed using random forests. Of the filtered metabolites, 883 were significantly differentially expressed in the CR groups when compared with the 12AL control group (Benjamini–Hochberg adjusted *p* ≤ .2; Supplementary Table 1).

**Figure 1.**
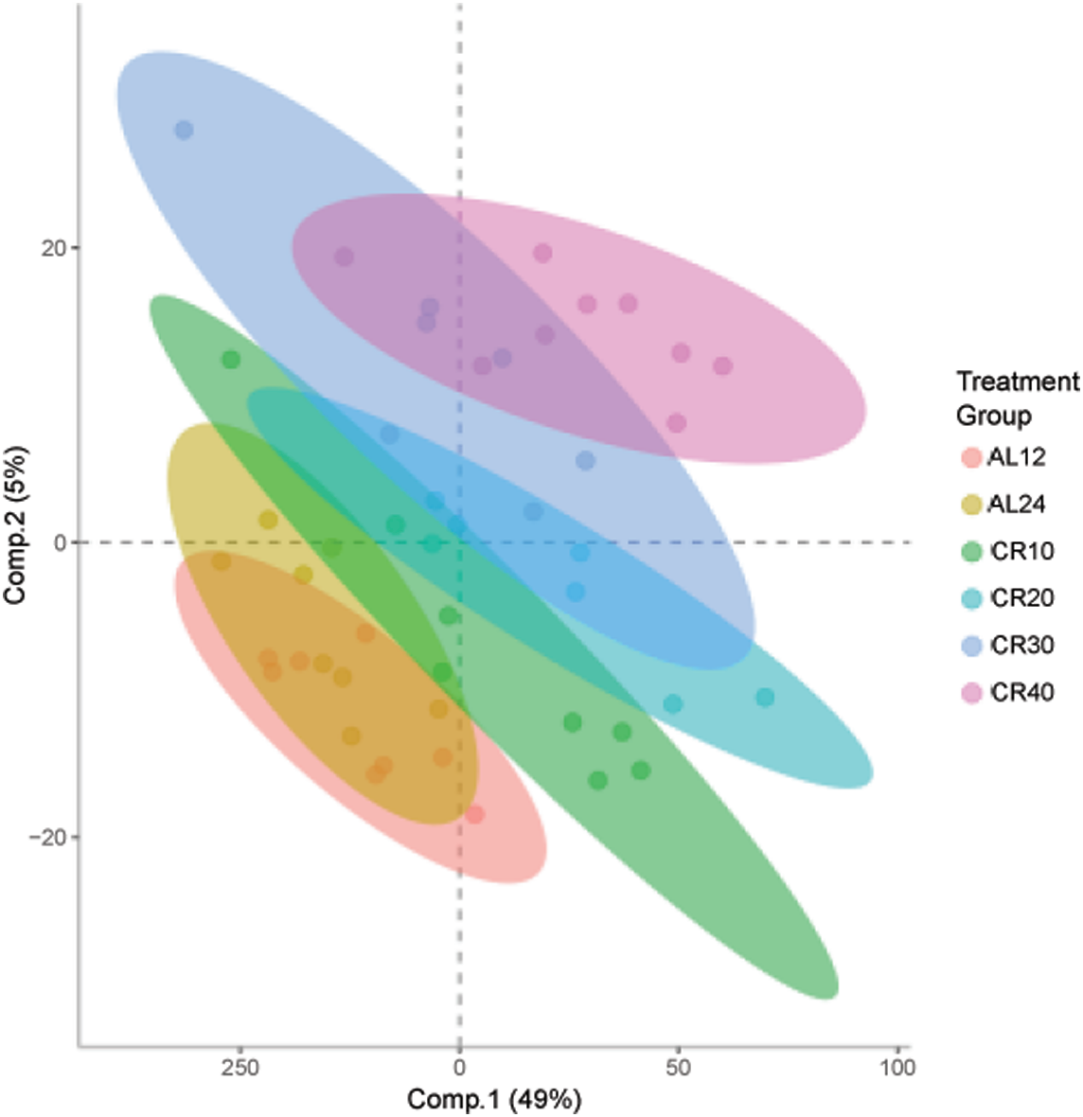
Orthogonal Partial Least Square Discriminate Analysis (O-PLS-DA) demonstrates the differentiation effect of diet in metabolomic profiles. The O-PLS-DA plot showed significant separation among samples on the basis of the model quality parameters: RX^2^, *Q*^2^, and root mean square error of prediction.

The O-PLS-DA model applied to all normalized identified and non-identified filtered metabolites (Figure 1) indicated that around 40% variance in the metabolites could be explained by the dietary treatment (15% for the first two components; single-sample *t* test between model parameters and permuted parameters *n* = 1,000, *p* < .001, RX^2^ = 42.60, *Q*^2^ = 0.953, root mean square error of prediction = 0.374). Of 2,876 *m*/*z* features, 755 features (≈26% of all metabolites) were judged to be significantly contributing to discrimination between treatment groups, using an FDR cutoff *p* value of less than .05. Of these, we were able to identify 257 metabolites (Supplementary Table 2). The model indicated that several fatty acids and membrane lipids (including glycerophosphoethanolamine, 3-oxododecanoic acid, sphingosine, linoleic acid, and lysophosphatidylcholine), molecules involved in DNA synthesis (uracil, adenosine, guanosine, cytosine, AMP, and NADPH), TCA cycle molecules (FAD, NAD+, and CoA), amino acids (l-alanine, l-phenylalanine, and s-adenosyl-l-methionine), and taurine and dopamine were important in discriminating the different CR groups.

### Pathway Analysis Indicated Changes in the TCA Cycle, Amino Acids, and Catecholamine Production

**Figure 2.**
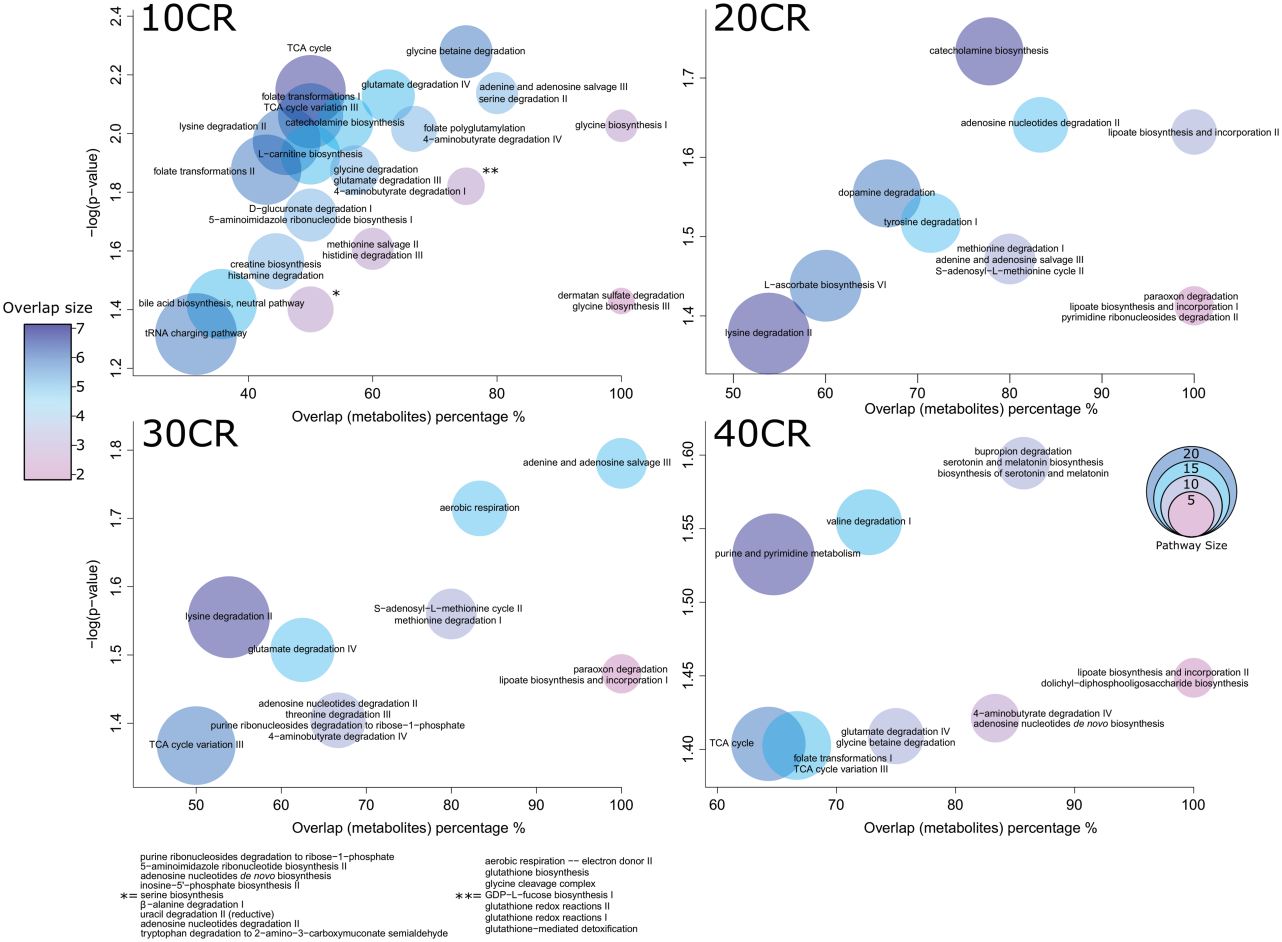
Bubble plots depicting significant pathways changed with *mummichog*. Percentage of metabolites overlapping with whole pathway on the x-axis and minus log *p* value on the y-axis. Size of the bubble indicates total pathway size and color indicates number of metabolites found. CR, calorie restriction. 10CR, 20CR, 30CR, 40CR: 10, 20, 30, 40% calorie restricted respectively. Relative to 12-hour ad libitum (12AL) access to food control.

We identified pathways in which the significantly differentiated metabolites were involved using both *mummichog* (Supplementary Table 3) and IPA (Supplementary Table 4). For *mummichog* analysis, we used unidentified filtered *m*/*z* values, alongside unadjusted *p* values from pairwise comparisons, fold changes relative to 12AL and retention times (57) (Figure 2). Only metabolites with KEGG or HMDB IDs were used in IPA, which resulted in 301 analysis ready metabolites, alongside their corresponding *p* values and log fold changes relative to 12AL. It is beneficial to use two different approaches to metabolic pathway enrichment as it removes inherent database biases. Although IPA relies on predetermined metabolite IDs and databases, *mummichog* predicts significant metabolic pathways using the collective power of all *m*/*z* features and potential networks (57).

There were significant changes in energy production, amino acid, nucleotide, hormone, and fatty acid pathways. Although components of TCA cycle pathways (Supplementary Table 3) in 40, 30, and 10CR (relative to 12AL) were mainly increased (*p* = .039, *p* = .043, and *p* = .007, respectively), the opposite was true for the 24AL group (relative to the 12AL group) in which most metabolites were decreased (*p* < .001). In addition to the TCA cycle, other energy production pathways were increased such as lipoate biosynthesis (Supplementary Table 3) at 40, 30, and 20CR (*p* = .036, *p* = .034, and *p* = .023, respectively), 4-aminobutyrate degradation (Supplementary Table 3) at 40, 30, and 10CR (*p* = .038, *p* = .039, and *p* = .010, respectively), NAD biosynthesis (Supplementary Table 4) at 40 and 20CR (*p* = .002, *p* = .011, respectively), folate transformations (Supplementary Table 3) at 40 and 10CR (*p* = .040 and *p* = .009, respectively), and the γ-glutamyl cycle (Supplementary Table 4) at 20 and 10CR (*p* = .019 and *p* = .010, respectively). Also increased were components of gluconeogenesis (Supplementary Table 4) at 40 and 20CR (*p* = .020 and *p* = .050) and glutamate degradation (glutamate feeds into the TCA cycle) at 40, 30, and 10CR (Supplementary Table 3; *p* = .039, *p* = .031, and *p* = .007, respectively), whereas at 24AL relative to 12AL, components were decreased (*p* = .001).

Changes in amino acids were widespread (*mummichog* results AL12: 12-hour ad libitum , AL24: 24-hour ad libitum access to food. CR10, CR20, CR30, CR40: 10, 20, 30, 40% calorie restricted respectively in Supplementary Table 3 and IPA in Supplementary Table 4). With increasing CR, the majority of pathways were increased, including threonine degradation (Supplementary Table 4) at 40 and 30CR (*p* = .001 and *p* = .003, respectively), phenylalanine degradation IV (Supplementary Table 4) at 40, 30, and 20CR (*p* = .001, *p* = .018, and *p* = .035, respectively), and glycine degradation (Supplementary Table 4) at 40, 30, 20, and 10CR (*p* = .015, *p* = .003, *p* = .005, and *p* < .001, respectively). Phenylalanine degradation I (Supplementary Table 4) was increased at 30 and 20CR (*p* = .003 and *p* = .005, respectively) but was unchanged at 40CR. Components of tryptophan degradation were increased (via three mechanisms) at 40CR (Supplementary Table 4; *p* = .002, *p* = .031, and *p* = .032) as were components of the serine and glycine biosynthesis superpathway (Supplementary Table 4; *p* = .044) and lysine degradation (*p* = .044). S-adenosyl-l-methionine cycle and methionine degradation components (Supplementary Table 4) were increased at 20 and 30CR (*p* = .028 and *p* = .034, respectively) whereas those of the tyrosine degradation pathway were mainly decreased at 20CR (Supplementary Table 3, *p* < .001). At 24AL relative to 12AL, glycine degradation, cysteine biosynthesis, and methionine degradation (Supplementary Table 4) were increased (*p* < .001 and *p* = .001, respectively). However, the superpathway of methionine degradation was decreased (*p* = .009), as was l-serine degradation (*p* = .039).

**Figure 3.**
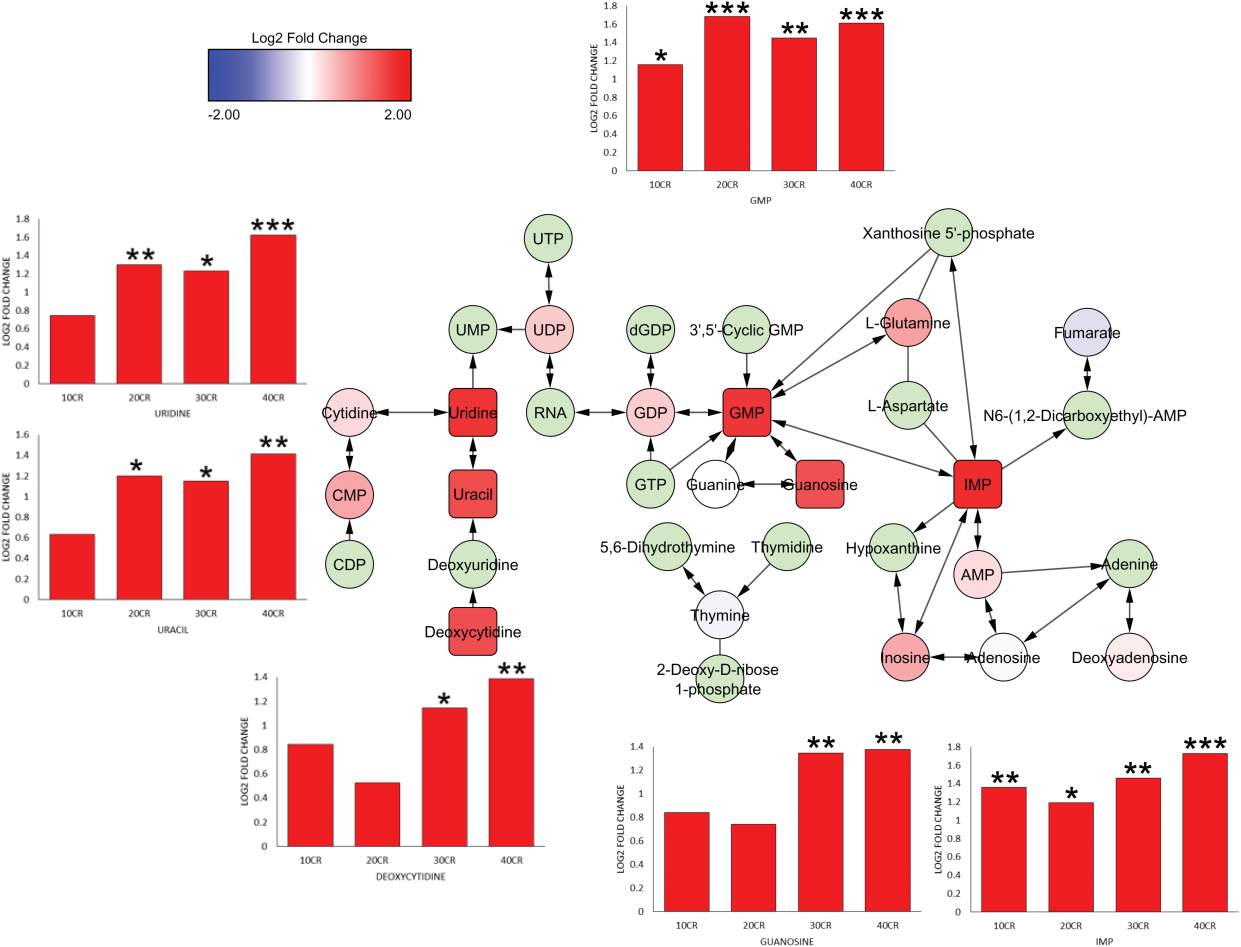
Metabolite network indicating the increase in nucleobases and nucleotides. Squares indicate significantly differentially expressed (SDE) metabolites. Graphs associated with SDE metabolites indicate increase in fold change at each calorie restriction level relative to 12AL. *p < .05, **p < .01, ***p < .001 (adjusted). 10CR, 20CR, 30CR, 40CR: 10, 20, 30, 40% calorie restricted respectively. AMP: adenosine monophosphate, CDP: cytidine diphosphate, CMP: cytidine monophosphate, GDP: guanosine diphosphate, GMP: guanosine monophosphate, GTP: guanosine triphosphate, IMP: inosine monophosphate, RNA: ribonucleic acid UDP: uridine diphosphate, UMP: uridine monophosphate, UTP: uridine triphosphate.

Purine, pyrimidine, nucleotide, and nucleoside components showed major increases with CR (Figure 3; Supplementary Tables 3 and 4). Specifically, components of adenosine nucleotide biosynthesis (Supplementary Table 3) were increased at 40CR (*p* = .038), adenine and adenosine salvage (Supplementary Table 3) were increased at 30, 20, and 10CR (*p* = .017, *p* = .034, and *p* = .007, respectively) and components of adenosine nucleotide degradation (Supplementary Table 4) were increased at 40, 30, 20, and 10CR (*p* = .003, *p* = .039, *p* = .023, and *p* = .040, respectively). The majority of purine and pyrimidine metabolism (Supplementary Table 3) components at 40CR (*p* = .029) and purine ribonucleosides degradation to ribose-1-phosphate (Supplementary Table 3) at 30 and 10CR (*p* = .039 and *p* = .040, respectively) were increased, while components of the tRNA charging pathway (Supplementary Table 3) were increased at 10CR (*p* = .048), whereas at 24AL relative to 12AL the majority of components in this pathway were decreased (*p* < .001).

**Figure 4.**
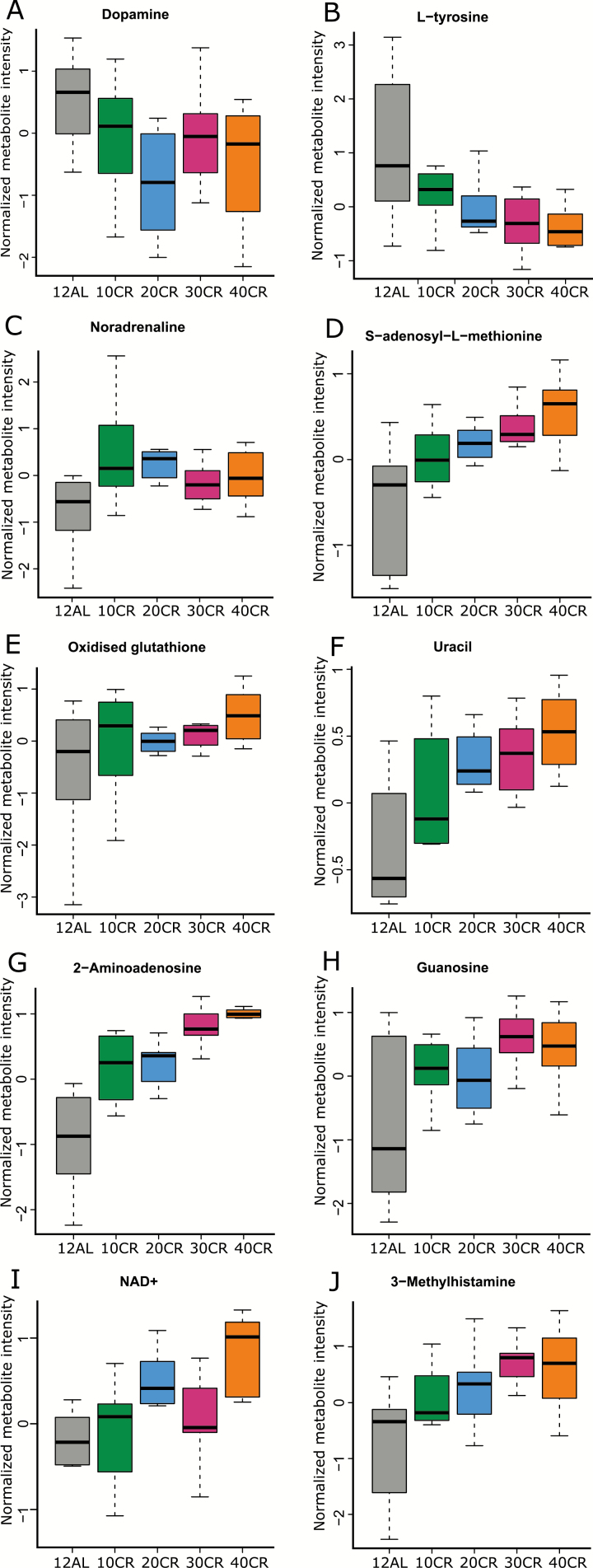
Metabolites potentially related to brown adipose tissue (BAT) activation that correlated significantly with the level of calorie restriction (CR; adjusted *p* ≤ .05). 12AL: 12-hour ad libitum access to food. 10CR, 20CR, 30CR, 40CR: 10, 20, 30, 40% calorie restricted respectively. A: Dopamine, B: L-tyrosine, C: Noradrenaline, D: S-adenosyl-L-methionine, E: Oxidised glutathione, F: Uracil, G: 2-aminoadenosine, H: Guanosine, I: NAD+ and J: 3-methylhistamine.

**Figure 5.**
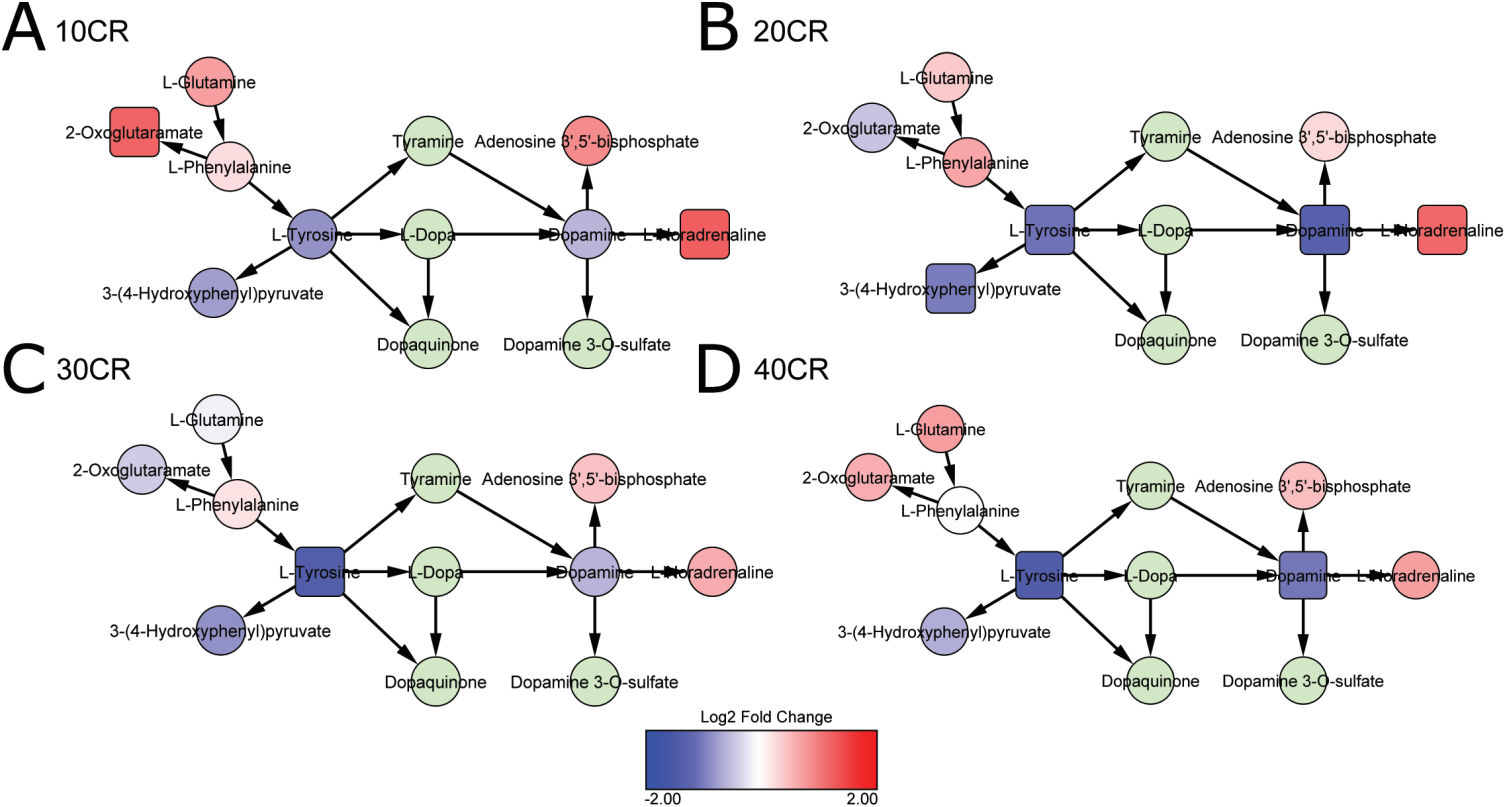
Metabolite networks resulting in upregulation of norepinephrine (l-noradrenaline) through decreasing l-tyrosine and dopamine. Significantly differentially expressed metabolites (adjusted *p* ≤ .05) represented as squares. 10CR, 20CR, 30CR, 40CR: 10, 20, 30, 40% calorie restricted respectively. Relative to 12-hour ad libitum (12AL) access to food control.

With CR, changes in catecholamine pathways indicate catecholamine production was increased (Supplementary Tables 3 and 4). Precursors tyrosine and dopamine were decreased (Figure 4A and B), whereas the downstream product noradrenaline was increased at all CR levels (Figures 4C and 5A–D). Components of noradrenaline and adrenaline degradation were increased at 20CR (*p* = .032) and catecholamine biosynthesis pathways were significant at 40, 30, 20, and 10CR and appeared to favor increases in catecholamines (Supplementary Table 4) from precursors (*p* = .003, *p* = .007, *p* < .001, and *p* = .008, respectively), as did the dopamine degradation pathway (Supplementary Table 4), which was significant at 40 and 20CR (*p* = .006 and *p* = .022, respectively), and l-DOPA degradation (Supplementary Table 4) at 40 and 20CR (*p* = .037 and *p* = .013, respectively). In addition, at 40CR, all components of serotonin and melatonin biosynthesis (Supplementary Table 3) were increased (*p* = .025), as was serotonin receptor signaling (Supplementary Table 4, *p* = .031). Surprisingly, catecholamine biosynthesis and dopamine degradation (Supplementary Table 4) were also upregulated at 24AL relative to 12AL (*p* = .009 and *p* = .013, respectively).

In addition, components of bile acid biosynthesis (Supplementary Table 4), taurine biosynthesis, l-carnitine biosynthesis, and sphingosine and sphingosine-1-phosphate metabolism pathways (Supplementary Table 4) were increased at 40CR (*p* = .003, *p* = .011, *p* = .031, and *p* = .031, respectively). An upregulation of similar pathways was also seen in metabolomics analyses of the liver (58) and plasma (59) of the same mice. This suggests that some of the metabolic remodeling that occurs during CR is systemic, including a shift from glycolysis to lipolysis (60).

### Metabolites Associated With Increasing CR

In the preceding section, we focused on the enrichment of pathways based on the significantly differentially expressed metabolites identified. Here, we turn our attention to metabolites that may potentially be associated with the life-span effects of CR. Because meta-analyses indicate that life span increases as level of CR increases, we correlated metabolites with level of CR to determine if any metabolites were positively or negatively associated (Supplementary Table 5; Figure 4). Correlations were corrected for type 1 errors using the Benjamini–Hochberg adjustment. Dopamine correlated negatively with level of CR (*R* = –.365, *p* = .031; Figure 4A), whereas the antioxidants s-adenosyl-l-methionine (Figure 4D) and oxidized glutathione (Figure 4E) correlated positively with CR (*R* = .509 and .222, *p* = .046 and .048, respectively). Several nucleic acids and other derivatives also correlated positively with CR including uracil (*R* = .478, *p* = .015; Figure 4F), 2-aminoadenisine (*R* = .867, *p* < .001; Figure 4G), guanosine (*R* = .544, *p* = .003; Figure 4H), and nicotinamide adenine dinucleotide (NAD+, *R* = .399, *p* = .003; Figure 4I). Although serotonin did not significantly correlate with level of CR, it is thought to be stored with histamine in BAT and 3-methylhistamine correlated positively with CR level (*R* = .470, *p* = .006; Figure 4J).

### Correlation of BAT Metabolites With Physical Parameters

**Figure 6.**
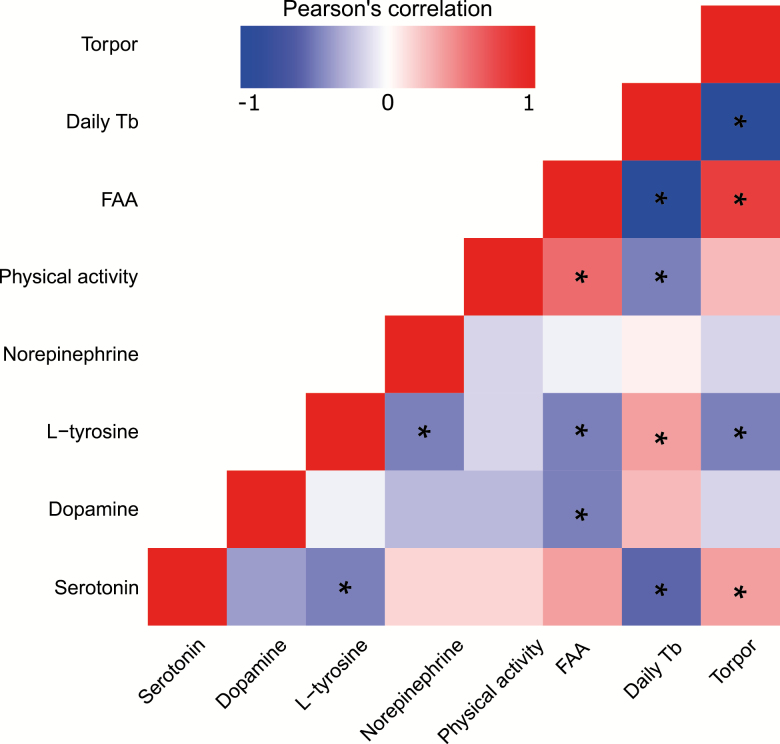
Correlation matrix depicting Pearson’s correlations between incidences of torpor, mean daily body temperature (Daily Tb), food anticipatory activity (FAA), physical activity over the final 2 weeks, and metabolites potentially involved in BAT activation, norepinephrine, l-tyrosine, dopamine, and serotonin. *p* values corrected for false discovery using Benjamini–Hochberg, * = *p* ≤ .05.

To further disentangle what potential roles metabolites may be playing in producing the beneficial effects of CR, we correlated metabolites levels against mean daily body temperature, FAA, and total physical activity, all calculated over the final 2 weeks of CR (Supplementary Table 5). Serotonin was negatively correlated with body temperature (*R* = –.454, *p* = .040), as was 2-aminoadenosine (*R* = –.816, *p* < .001). 3-Methylhistamine, uracil, 2-aminoadenosine, and guanosine correlated positively with FAA (*R* = .536, .449, .864, and .623, respectively; and *p* = .014, .049, <.001, and .009, respectively). We also correlated potential BAT activating metabolites and physical parameters that had been previously reported in the same mice; these included incidence of torpor, daily average body temperature (28), physical activity, FAA (29), norepinephrine, l-tyrosine, dopamine, and serotonin (Figure 6). We found no significant correlation between norepinephrine and dopamine. However, norepinephrine was significantly negatively correlated with the level of its precursor, l-tyrosine (*R* = –.38, *p* = .009).

### Pathways Potentially Associated With Life span

We used the coefficients of those metabolites that were significantly correlated with CR level to determine potential metabolic pathways that may be related to life span (Supplementary Table 6). Metabolites were correlated with the level of CR, and resulting correlation coefficients and *p* values were used for pathway enrichment in *mummichog*. The majority of pathways found appeared positively associated with level of CR, except for the tryptophan degradation I pathway (*p* = .033) that was negatively associated and the tryptophan degradation pathway to 2-amino-3-carboxymuconate semialdehyde pathway, which had the same number of positively and negatively correlated metabolites (*p* = .039). Among those positively associated pathways were nucleotide pathways such as adenine and adenosine salvage, purine riconucleoside degradation and adenosine nucleotide degradation (*p* = .015, *p* = .018, and *p* = .039, respectively). In addition to this, glutamate degradation, TCA cycle, and dopamine degradation pathways were positively associated with potential life span (*p* = .017, *p* = .035, and *p* = .043, respectively).

## Discussion

Mice undergoing CR, particularly higher levels of CR, face a dilemma of resource allocation. Although after approximately a month, most available tissue for energy has been broken down and body mass stabilizes, animals must still strike a balance between energy expenditure and energy conservation (30). Activation of BAT tissue under CR, therefore, is somewhat paradoxical, as it notoriously increases energy expenditure and uncouples the electron transport chain to produce heat. Previously it was suggested that C57BL/6 mice on 40% CR had elevated “beigeing” in subcutaneous and visceral WAT, possibly through type 2 cytokine signaling and increased sirtuin 1 (SIRT1) in M2 macrophages in fat tissues (23). However, recent work has indicated that alternate macrophage activation does not induce adaptive thermogenesis through production of catecholamines (61), and the same mice used in this study did not show any increase in UCP1 messenger RNA in epididymal WAT (62).

In the BAT, we found that NAD+ increased with increasing CR, which may directly reflect energy availability. In addition to its role as a co-substrate in the TCA cycle, NAD+ acts as a direct reflection of energy reserves in the cell, and can, therefore, act as sensor of energy stress to other molecules, such as the NAD+-dependent deacetylase SIRT1 (63). Unfortunately, we did not have measures of SIRT1 activation to confirm such an effect. As SIRT1 and its orthologes have been reported to increase with CR in a variety of species and have also been associated with increasing life span in yeast, worms, and flies (64–66), the increase in NAD+ and potential increase in SIRT1 activation may contribute to the linear relationship between CR and life span. However, opposing data indicated that overexpression did not increase life span in either worms or flies, leaving the role of SIRT1 in aging unclear (67). Whole-body SIRT1 expression in mice has previously been shown to reduce the onset of cancer although its role in life span increase remains unclear (68). Interestingly, tissue-specific overexpression of SIRT1 in the brain has been shown to increase longevity in mice (69), suggesting more localized increases in SIRT1, such as in the BAT, may be beneficial.

Although we observed a decrease in BAT mass with CR, it was far more conserved than other fat depots (30). It is possible that mice retain BAT to facilitate heat production in times of cold stress due to loss of WAT and to assist with rewarming after periods of torpor (3). In an earlier article using the same mice, we showed that CR reduced body temperature during the resting phase of the circadian cycle (28), and BAT may need to be activated to enable them to increase their body temperature on waking. At 40CR, mice increased temperature from 22 to 37°C in 2–3 hours (28).

When thermogenesis is required, BAT is activated in several ways, the best studied being through the sympathetic nervous system-mediated release of norepinephrine (3). Amino acid levels affect catecholamine production, and tyrosine is a precursor for dopamine, which in turn is a precursor for norepinephrine (70). In BAT, we found that l-tyrosine and dopamine were significantly decreased at all levels of CR (dopamine was decreased linearly). This may be because no dopamine storage structure has been found in BAT, and it is converted to norepinephrine (71). We found that norepinephrine was significantly negatively correlated with its precursor l-tyrosine (via dopamine), which might indicate a shift toward BAT activation. Although norepinephrine is the most powerful stimulator of BAT, additional mechanisms may be acting to stimulate thermogenesis. We found that serotonin, which can activate BAT and cause browning of WAT (72), was also increased with CR. In addition, in BAT serotonin is stored within mast cells with histamine (71) and we found that 3-methylhistamine, for which histamine is a substrate, was increased with CR.

Uncoupling of the electron transport chain decreases the efficiency of mitochondrial energy production and therefore requires a significant increase in substrates. Hence, UCP1 activation must galvanize several mechanisms at once (73). Norepinephrine interaction with β3 adrenergic receptors results in triacylglycerol hydrolysis (74), and resultant fatty acids are then taken up by mitochondria for β-oxidation and UCP1 activation (75). In rodents, BAT is a major lipid clearing organ and the primary source of triacylglycerols for BAT activation circulate in the plasma (76). In male C57BL/6 mice, injecting UCP1 adenovirus vector into epididymal WAT improves whole-body glucose tolerance and insulin sensitivity through clearance of fats and glucose from the plasma (77). In the BAT metabolome under CR, we found that lipid and glucose pathways increased. These pathways are potentially being activated to accommodate elevated mitochondrial uncoupling that follows increased activation of UCP1. Unfortunately, because the tissue was used for metabolomics we do not have simultaneous measures of transcriptomic or proteomic responses to verify changes in the levels of key uncoupling proteins including UCP1. Hence, we cannot distinguish whether the changes in the metabolite levels are secondary to changes in UCP1 levels or primary responses to the levels of CR.

Clearance of both lipids and glucose from the plasma by UCP1 stimulation has many implications for health, including reduction in the risk of type 2 diabetes, obesity, and cardiovascular disease (78,79). Of the pathways potentially associated with life span in the BAT by virtue of their relationship to the level of restriction, we found that upregulation of the TCA cycle was significant. Induction of BAT and beiging has also been shown to improve glucose tolerance and promote increased insulin sensitivity (80), benefits often associated with CR (81,82).

Uncoupling of the electron transport chain has previously been associated with longevity (83). Previous work has indicated that mitochondrial uncoupling has beneficial effects in male (84) and female mice (85), which is thought to reduce reactive oxygen species production and improve the antioxidant defense system resulting in improved longevity and physiological and metabolic health. However, previous work in the liver of these mice indicated that although CR has no effect on oxidative, DNA, protein, or lipid damage after 3 months, it did lower antioxidant activity of catalase. Furthermore, major urinary proteins, a marker of reproductive investment that may also accrue oxidative damage, showed a significant downward trend with increasing CR (82). A concomitant increase in antioxidant capacity with mitochondrial uncoupling may be tissue specific, as previous work has shown an increase in both catalase and superoxide dismutase and redox signaling in UCP1 transgenic mice (84). Despite the apparent lowering of antioxidant capacity in the liver, in the BAT we see an increase in the production of potentially beneficial metabolites. In our mice, both s-adenosyl-l-methionine and oxidized glutathione were significantly positively associated with level of CR. An increase in oxidation of glutathione has been seen previously in a metabolomic analysis of acute cold exposure in BAT of C57BL/6 mice (36). This may have longer lasting implications for overall health of the tissue and reduction in age-associated breakdown of cells.

In our previous work on the mice in this study, we showed that mice had reduced insulin, fasting glucose, and adiposity (82) and that in part, this energy balance was maintained through reduction in mean body temperature, which stabilized around day 30, but continued to decline after day 70 (28). Body temperature declined particularly during lights on, when animals were sleeping, and in some cases animals entered periods of torpor. Previous work has indicated that a relationship between BAT and torpor exists, in *ob*/*ob* mice, which can spontaneously enter torpor due to a lack of leptin, food restriction has been shown to increase both torpor and the capacity for thermogenesis in BAT (86). We found that after periods of torpor, body temperature rose dramatically in concordance with a surge in physical activity or FAA (28). In fact, in the few hours before being fed, FAA accounted for 38.6% of total daily activity in the last 20 days at 40CR (29). Facilitation of these high activity levels may be occurring through acute activation of BAT to increase body temperature before active periods. It has been hypothesized that FAA mimics foraging behavior to protect against starvation when resources are scarce (87). We showed that a positive correlation exists between FAA and the precursors of DNA or messenger RNA, indicating a potential connection between awakening from torpor and induction of cell proliferation and protein production (eg. production of UCP1). Mice undergoing CR need to maximize conservation of energy, and low body temperature may facilitate this. However, to maximize foraging potential, and thereby increase calorie intake, BAT activation may be used. As we found, mouse weight stabilizes after 1 month. It appears this trade-off is maintained, potentially in addition to the suppression of basal metabolic rate, although this effect disappeared when corrected for body weight (30,88).

**Figure 7.**
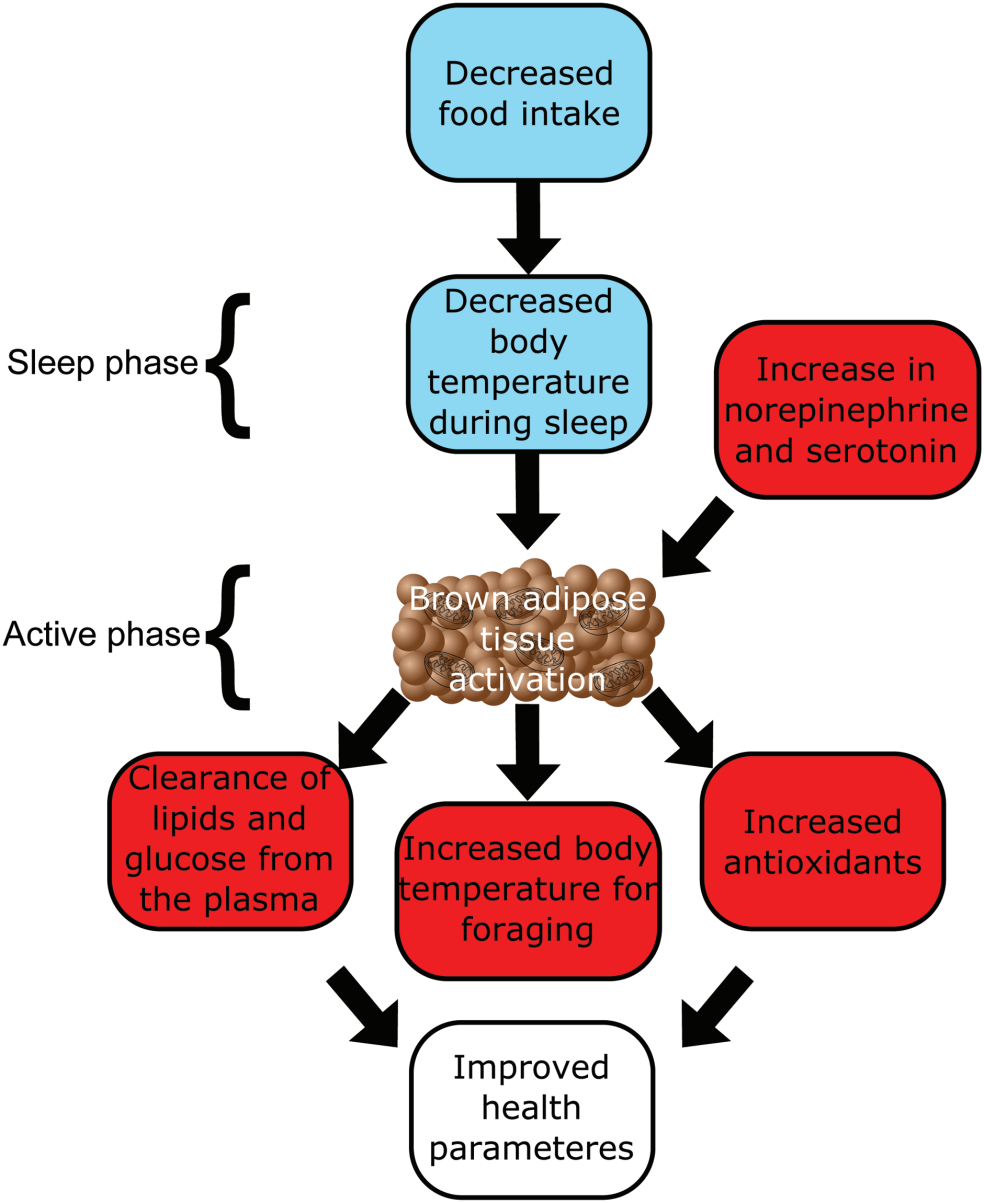
Concept diagram indicating the effects of calorie restriction on brown adipose tissue.

The metabolic changes in BAT observed in this study may indicate that BAT activation, potentially through UCP1, and acute thermogenesis may occur in our CR animals. A big limitation of this study, in common with most other studies of metabolomics, is that steady state snap-shots of the levels of metabolites may be a poor reflection of metabolite flux. Yet, flux may be much more revealing concerning the metabolic responses to CR. It is also important to note that directionality or causality cannot be inferred from metabolomics alone, and additional investigation is required to determine the functional roles of the observed changes. We can infer, however, that due to the effect of CR on body temperature and FAA that thermogenesis plays a part in this acute temperature increase. From an evolutionary standpoint, this is consistent, as animals undergoing times of food shortage would need to conserve energy during the day, but be able to become active quickly to spend as much time foraging as possible during the night. Stimulation of acute thermogenesis may provide lasting beneficial effects in these animals, including those that increase longevity, such as clearance of lipids and glucose from the blood and increased antioxidant defense. Importantly, these effects were seen after only 3 months of CR, which indicating remodeling of BAT metabolism occurs rapidly on food restriction. In addition, body mass in these mice stabilized after approximately 30 days (30). Therefore, though BAT was used to an extent, over half of the BAT was conserved even at the highest level of restriction. This indicates an important role for thermogenesis in restricted mice. Owing to the relationship between BAT, glucose, insulin, and lipids, this work could have implications for the relief of metabolic disorders far beyond diabetes, including atherosclerosis and cardiovascular disease (Figure 7).

## Data Accessibility

Work toward having all the data from this series of articles online is currently ongoing. All significant metabolites in relation to CR manipulation are listed in Supplementary Material 1. Data on the nonsignificant metabolites are freely available for anyone who requests it from the corresponding author at j.speakman@abdn.ac.uk.

## Funding

The work was supported by the UK Biotechnology and Biological Sciences Research Council BBSRC (BB/G009953/1, BB/P009875/1 and BB/J020028/1 to J.R.S.) and a studentship of C.L.G. from the BBSRC EastBio Doctoral Training Partnership (1438803). C.L.G. received support from the laboratory of D.E.L.P.; D.E.L.P was supported in part by National Institute of Health grant AGO49494.

## Conflict of Interest

The authors declare no conflicts of interests.

## Supplementary Material

glz023_suppl_Supplementary_Material-1

glz023_suppl_Supplementary_Tables-1-6
