# Supplementary material for: The Effects of Graded Levels of Calorie Restriction: XIV. Global Metabolomics Screen Reveals Brown Adipose Tissue Changes in Amino Acids, Catecholamines, and Antioxidants After Short-Term Restriction in C57BL/6 Mice
Source: J Gerontol A Biol Sci Med Sci. 2019 Apr 9;75(2):218–29. doi: 10.1093/gerona/glz023 (PMC7530471; doi:10.1093/gerona/glz023)
Supplement: glz023_suppl_Supplementary_Tables-1-6 [file glz023_suppl_supplementary_tables-1-6.docx]

**Supplementary Tables for The effects of graded levels of calorie restriction: XIV. Global metabolomics screen reveals brown adipose tissue changes in amino acids, catecholamines and antioxidants after short-term restriction in C57BL/6 mice.**

### Supplementary Table 1: Number of significantly differentially expressed (SDE) metabolites, relative to 12AL control for each CR treatment level and the 24AL group (Benjamini Hochberg adjusted P ≤ 0.2).

|  | 24AL | 10CR | 20CR | 30CR | 40CR |
| --- | --- | --- | --- | --- | --- |
| Increased | 0 | 0 | 151 | 118 | 442 |
| Unchanged | 2876 | 2876 | 2644 | 2697 | 2082 |
| Decreased | 0 | 0 | 81 | 61 | 352 |

### Supplementary Table 2: Metabolites identified in O-PLS-DA model to be significantly discriminating between treatment groups. Pcorr = partial correlation coefficient.

| Metabolite | Model weight | Pcorr | Loadings | P-value |
| --- | --- | --- | --- | --- |
| Aminoadipic acid | -0.015 | 0.872 | -0.015 | 0.000 |
| PC(14:020:2(11Z14Z)) | -0.029 | 0.844 | -0.029 | 0.000 |
| Pyrimidodiazepine | -0.019 | 0.795 | -0.019 | 0.000 |
| CDP-ethanolamine | -0.016 | 0.789 | -0.016 | 0.000 |
| Guanosine monophosphate | -0.019 | 0.789 | -0.019 | 0.000 |
| Cinncassiol C3 | -0.004 | 0.781 | -0.004 | 0.000 |
| Fluo-3 | -0.012 | 0.767 | -0.012 | 0.000 |
| 1-nonadecanoyl-2-(7Z10Z13Z16Z-docosatetraenoyl)-glycero-3-phosphate | -0.025 | 0.763 | -0.025 | 0.000 |
| O-Phosphoethanolamine | -0.023 | 0.760 | -0.023 | 0.000 |
| NAD | -0.023 | 0.748 | -0.023 | 0.000 |
| Clomeprop | -0.021 | 0.745 | -0.021 | 0.000 |
| Cyclic ADP-ribose | -0.023 | 0.743 | -0.023 | 0.000 |
| L-Valine | -0.012 | 0.742 | -0.012 | 0.000 |
| FAD | -0.022 | 0.740 | -0.022 | 0.000 |
| CDP-choline | -0.011 | 0.738 | -0.011 | 0.000 |
| 7-Mercaptoheptanoic acid | -0.016 | 0.738 | -0.016 | 0.000 |
| (-)-Epigallocatechin | -0.014 | 0.727 | -0.014 | 0.000 |
| GDP-4-keto-6-L-deoxygalactose | -0.013 | 0.726 | -0.013 | 0.000 |
| 4-Amino-2-hydroxylamino-6-nitrotoluene | -0.009 | 0.723 | -0.009 | 0.000 |
| PC(14:020:3(5Z8Z11Z)) | -0.024 | 0.723 | -0.024 | 0.000 |
| LysoPC(16:0) | -0.025 | 0.723 | -0.025 | 0.000 |
| NA | -0.016 | 0.715 | -0.016 | 0.000 |
| GW 4064 | -0.021 | 0.713 | -0.021 | 0.000 |
| Uridine | -0.020 | 0.707 | -0.02 | 0.000 |
| N-(hexadecanoyl)-sphing-4-enine-1-phosphate | -0.011 | 0.700 | -0.011 | 0.000 |
| Dephospho-CoA | -0.013 | 0.693 | -0.013 | 0.000 |
| Uracil | -0.020 | 0.692 | -0.02 | 0.000 |
| NADPH | -0.012 | 0.691 | -0.012 | 0.000 |
| NA | -0.019 | 0.690 | -0.019 | 0.000 |
| AMP | -0.026 | 0.688 | -0.026 | 0.000 |
| Doxefazepam | -0.025 | 0.687 | -0.025 | 0.000 |
| Queuine | -0.010 | 0.685 | -0.01 | 0.000 |
| 3-Oxododecanoic acid | -0.012 | 0.684 | -0.012 | 0.000 |
| Valerylcarnitine | -0.012 | 0.680 | -0.012 | 0.000 |
| Oxidized glutathione | -0.016 | 0.676 | -0.016 | 0.000 |
| LysoPC(20:5(5Z8Z11Z14Z17Z)) | -0.027 | 0.675 | -0.027 | 0.000 |
| Heptopargil | -0.014 | 0.667 | -0.014 | 0.000 |
| 3-(Imidazol-4-yl)-2-oxopropyl phosphate | -0.017 | 0.665 | -0.017 | 0.000 |
| Cyclic-320-bis(12-ethanediyl acetal)-11alpha-(acetyloxy)-5alpha6alpha-epoxypregnane-320-dione | -0.020 | 0.665 | -0.02 | 0.000 |
| N-Acetyl-D-glucosamine | -0.009 | 0.664 | -0.009 | 0.000 |
| Delphinidin 3-(6-p-coumarylglucoside) | -0.013 | 0.662 | -0.013 | 0.000 |
| Stealthin C | -0.025 | 0.656 | -0.025 | 0.000 |
| S-Adenosyl-L-methionine | -0.021 | 0.655 | -0.021 | 0.000 |
| (-)-Menthyl acetate | -0.012 | 0.651 | -0.012 | 0.000 |
| LysoPC(18:1(9Z)) | -0.019 | 0.650 | -0.019 | 0.000 |
| Isoniazid alpha-ketoglutaric acid | -0.021 | 0.648 | -0.021 | 0.000 |
| Dihydrolipoate | -0.017 | 0.644 | -0.017 | 0.000 |
| 9-Hydroxy-2-nitrofluorene | -0.014 | 0.643 | -0.014 | 0.000 |
| Gibberellin A34-catabolite | -0.014 | 0.643 | -0.014 | 0.000 |
| 1-(5Z8Z11Z14Z17Z-eicosapentaenoyl)-glycero-3-phospho-(1-myo-inositol) | -0.023 | 0.641 | -0.023 | 0.000 |
| PI(16:022:4(10Z13Z16Z19Z)) | -0.028 | 0.641 | -0.028 | 0.000 |
| Cyclamate | -0.010 | 0.637 | -0.01 | 0.000 |
| Clobenpropit | -0.023 | 0.637 | -0.023 | 0.000 |
| NA | -0.010 | 0.636 | -0.01 | 0.000 |
| Pantetheine | -0.010 | 0.636 | -0.01 | 0.000 |
| Allamandin | -0.024 | 0.629 | -0.024 | 0.000 |
| alpha-D-glucopyranosyl 25-hydroxyhexacosanoate | -0.008 | 0.627 | -0.008 | 0.000 |
| Inosine | -0.018 | 0.627 | -0.018 | 0.000 |
| Megalomicin A | -0.021 | 0.626 | -0.021 | 0.000 |
| 4-Hydroxy-2-butynal | -0.017 | 0.625 | -0.017 | 0.000 |
| S-Nitrosoglutathione | -0.014 | 0.624 | -0.014 | 0.000 |
| Niacinamide | -0.019 | 0.623 | -0.019 | 0.000 |
| CMP | -0.020 | 0.623 | -0.02 | 0.000 |
| Cucurbitacin P | -0.023 | 0.622 | -0.023 | 0.000 |
| D-Lysopine | -0.014 | 0.622 | -0.014 | 0.000 |
| Terpendole E | -0.023 | 0.622 | -0.023 | 0.000 |
| GDP | -0.021 | 0.612 | -0.021 | 0.000 |
| Stealthin C | -0.027 | 0.610 | -0.027 | 0.000 |
| sn-Glycero-3-phosphoethanolamine | -0.010 | 0.610 | -0.01 | 0.000 |
| 5-Tetradecenoic acid | -0.018 | 0.608 | -0.018 | 0.000 |
| Anisomycin | -0.013 | 0.599 | -0.013 | 0.000 |
| Biotin | -0.023 | 0.596 | -0.023 | 0.000 |
| N-Acetylglucosamine 6-sulfate | -0.017 | 0.595 | -0.017 | 0.000 |
| 1-(11Z14Z-eicosadienoyl)-2-(4Z7Z10Z13Z16Z19Z-docosahexaenoyl)-glycero-3-phosphoserine | -0.022 | 0.595 | -0.022 | 0.000 |
| Actinorhodin | -0.010 | 0.591 | -0.01 | 0.000 |
| 2-Maleylacetate | -0.019 | 0.587 | -0.019 | 0.000 |
| epi-Tulipinolide diepoxide | -0.017 | 0.587 | -0.017 | 0.000 |
| PI(18:2(9Z12Z)16:0) | -0.023 | 0.587 | -0.023 | 0.000 |
| (9S10E12Z15Z)-9-Hydroxy-101215-octadecatrienoic acid | -0.014 | 0.583 | -0.014 | 0.000 |
| 1-Methylhistidine | -0.018 | 0.583 | -0.018 | 0.000 |
| 1-(1Z-hexadecenyl)-sn-glycero-3-phosphoethanolamine | -0.022 | 0.579 | -0.022 | 0.000 |
| Trihomomethionine | -0.009 | 0.578 | -0.009 | 0.000 |
| Kinetin | -0.010 | 0.577 | -0.01 | 0.000 |
| PC(14:022:5(4Z7Z10Z13Z16Z)) | -0.025 | 0.574 | -0.025 | 0.000 |
| Kobusone | -0.014 | 0.567 | -0.014 | 0.000 |
| 1-O-(1Z-hexadecenyl)-2-(14-hydroxy-4Z7Z10Z12E16Z19Z-docosahexaenoyl)-sn-glycero-3-phosphoethanolamine | -0.019 | 0.565 | -0.019 | 0.000 |
| 5-Methylcytidine | -0.017 | 0.563 | -0.017 | 0.000 |
| Propanoyl phosphate | -0.021 | 0.562 | -0.021 | 0.000 |
| Cysteinylglycine | -0.022 | 0.561 | -0.022 | 0.000 |
| Estra-135(10)16-tetraen-3-ol benzoate | -0.012 | 0.561 | -0.012 | 0.000 |
| 3-Butyn-1-ol | -0.012 | 0.559 | -0.012 | 0.000 |
| Kinobscurinone | -0.026 | 0.555 | -0.026 | 0.000 |
| Saphenic acid methyl ester | -0.017 | 0.554 | -0.017 | 0.000 |
| 2-Aminoadenosine | -0.012 | 0.553 | -0.012 | 0.000 |
| Isobutyryl-L-carnitine | -0.009 | 0.553 | -0.009 | 0.000 |
| S-Glutathionyl-L-cysteine | -0.019 | 0.550 | -0.019 | 0.000 |
| Prothioconazole | -0.013 | 0.547 | -0.013 | 0.001 |
| 1-(11Z14Z-eicosadienoyl)-2-(7Z10Z13Z16Z-docosatetraenoyl)-glycero-3-phosphoserine | -0.022 | 0.547 | -0.022 | 0.001 |
| Adenosine | -0.019 | 0.547 | -0.019 | 0.001 |
| PC(14:018:2(9Z12Z)) | -0.018 | 0.545 | -0.018 | 0.001 |
| Proacacipetalin | -0.020 | 0.543 | -0.02 | 0.001 |
| D-Alanyl-D-alanine | -0.024 | 0.540 | -0.024 | 0.001 |
| 1-(9Z12Z-octadecadienoyl)-glycero-3-phosphoethanolamine | -0.022 | 0.540 | -0.022 | 0.001 |
| Paspaline B | -0.015 | 0.539 | -0.015 | 0.001 |
| Cytosine | -0.013 | 0.535 | -0.013 | 0.001 |
| Riboflavin | -0.017 | 0.532 | -0.017 | 0.001 |
| 2-Oxoglutaramate | -0.021 | 0.529 | -0.021 | 0.001 |
| L-Malic acid | -0.024 | 0.529 | -0.024 | 0.001 |
| L-Phenylalanine | -0.019 | 0.525 | -0.019 | 0.001 |
| N-Formyl-4-amino-5-aminomethyl-2-methylpyrimidine | -0.014 | 0.524 | -0.014 | 0.001 |
| 2-Hydroxy-6-oxo-6-(2-hydroxyphenoxy)-hexa-24-dienoate | -0.018 | 0.524 | -0.018 | 0.001 |
| Phenylethylmalonamide | -0.022 | 0.522 | -0.022 | 0.001 |
| 2-Hydroxyfelbamate | -0.014 | 0.521 | -0.014 | 0.001 |
| PS(18:018:2(9Z12Z)) | -0.022 | 0.520 | -0.022 | 0.001 |
| 1-(9Z-nonadecenoyl)-2-(4Z7Z10Z13Z16Z19Z-docosahexaenoyl)-glycero-3-phosphocholine | -0.026 | 0.519 | -0.026 | 0.001 |
| Molybdopterin | -0.015 | 0.518 | -0.015 | 0.001 |
| 1-(6Z9Z12Z15Z-octadecatetraenoyl)-2-(7Z10Z13Z16Z-docosatetraenoyl)-glycero-3-phosphoserine | -0.014 | 0.515 | -0.014 | 0.001 |
| Cytidine | -0.017 | 0.513 | -0.017 | 0.001 |
| LysoPC(14:0) | -0.017 | 0.511 | -0.017 | 0.001 |
| 3-(12-hydroxyoctadecanoyl)oxy-4-(trimethylazaniumyl)butanoate | -0.014 | 0.511 | -0.014 | 0.001 |
| Terbufos | -0.023 | 0.510 | -0.023 | 0.001 |
| Guanosine | -0.011 | 0.510 | -0.011 | 0.002 |
| L-Glutamic acid | -0.023 | 0.509 | -0.023 | 0.002 |
| Methylimidazoleacetic acid | -0.016 | 0.507 | -0.016 | 0.002 |
| 4-Hydroxy-4-methylglutamate | -0.017 | 0.507 | -0.017 | 0.002 |
| Taurine | -0.024 | 0.506 | -0.024 | 0.002 |
| Norepinephrine | -0.020 | 0.505 | -0.02 | 0.002 |
| Nifuradene | -0.022 | 0.505 | -0.022 | 0.002 |
| 44-Diaminostilbene dihydrochloride | -0.020 | 0.505 | -0.02 | 0.002 |
| 3-(3-hydroxy-3-methylbutanoyl)oxy-4-(trimethylazaniumyl)butanoate | -0.020 | 0.502 | -0.02 | 0.002 |
| unsym-Bis(4-chlorophenyl)ethylene | -0.022 | 0.502 | -0.022 | 0.002 |
| Pymetrozine | -0.011 | 0.496 | -0.011 | 0.002 |
| Makisterone B | -0.015 | 0.493 | -0.015 | 0.002 |
| LysoPC(18:0) | -0.023 | 0.489 | -0.023 | 0.003 |
| Nodakenin | -0.020 | 0.488 | -0.02 | 0.003 |
| (2-aminoethoxy)(2R)-2-(9Z)-hexadec-9-enoyloxy-3-hydroxypropoxyphosphinic acid | -0.023 | 0.487 | -0.023 | 0.003 |
| NA | -0.013 | 0.482 | -0.013 | 0.003 |
| Buprenorphine | -0.012 | 0.481 | -0.012 | 0.003 |
| S-(Hydroxyphenylacetothiohydroximoyl)-L-cysteine | -0.011 | 0.481 | -0.011 | 0.003 |
| L-tryptophan | -0.013 | 0.481 | -0.013 | 0.003 |
| 3-Methylglutaconic acid | -0.012 | 0.478 | -0.012 | 0.003 |
| 12-di-(9Z-hexadecenoyl)-sn-glycero-3-phospho-(1-myo-inositol) | -0.025 | 0.477 | -0.025 | 0.004 |
| Thioacetamide | -0.014 | 0.474 | -0.014 | 0.004 |
| 1-tridecanoyl-sn-glycero-3-phosphocholine | -0.025 | 0.472 | -0.025 | 0.004 |
| Neocnidilide | -0.016 | 0.471 | -0.016 | 0.004 |
| 2-Dehydro-D-xylonate | -0.013 | 0.470 | -0.013 | 0.004 |
| Dimethyl hydrogen phosphite | -0.027 | 0.470 | -0.027 | 0.004 |
| SM(d18:016:1(9Z)) | -0.024 | 0.469 | -0.024 | 0.004 |
| Inosinic acid | -0.015 | 0.467 | -0.015 | 0.005 |
| Aflatoxin B1-endo-89-epoxide | -0.019 | 0.465 | -0.019 | 0.005 |
| Methyl 1818-dibromo-17-octadecen-57-diynoate | -0.012 | 0.463 | -0.012 | 0.005 |
| Xanthopterin-B2 | -0.016 | 0.463 | -0.016 | 0.005 |
| PS(16:018:2(9Z12Z)) | -0.024 | 0.462 | -0.024 | 0.005 |
| Terpendole G | -0.026 | 0.461 | -0.026 | 0.005 |
| Neuraminic acid | -0.020 | 0.458 | -0.02 | 0.006 |
| Aminoparathion | -0.012 | 0.458 | -0.012 | 0.006 |
| N-Acetyl-L-histidine | -0.018 | 0.455 | -0.018 | 0.006 |
| 8-Hydroxyguanine | -0.025 | 0.453 | -0.025 | 0.006 |
| Coenzyme B | -0.018 | 0.452 | -0.018 | 0.007 |
| 25-Furandicarboxylic acid | -0.017 | 0.446 | -0.017 | 0.008 |
| PS(18:020:4(8Z11Z14Z17Z)) | -0.028 | 0.445 | -0.028 | 0.008 |
| PI(16:020:2(11Z14Z)) | -0.021 | 0.445 | -0.021 | 0.008 |
| Oxaloglutarate | -0.013 | 0.443 | -0.013 | 0.008 |
| alpha-EMTBL | -0.024 | 0.443 | -0.024 | 0.008 |
| Fexofenadine | -0.021 | 0.442 | -0.021 | 0.008 |
| 3-deoxy-D-erythro-hex-2-ulosonic acid | -0.025 | 0.442 | -0.025 | 0.008 |
| 12-di-(4Z7Z10Z13Z16Z19Z-docosahexaenoyl)-sn-glycero-3-phosphocholine | -0.023 | 0.440 | -0.023 | 0.009 |
| Linoleic acid | -0.021 | 0.440 | -0.021 | 0.009 |
| Biotin | -0.016 | 0.437 | -0.016 | 0.009 |
| Sphingosine | -0.020 | 0.434 | -0.02 | 0.010 |
| PI(16:016:1(9Z)) | -0.024 | 0.433 | -0.024 | 0.010 |
| Nicotinuric acid | -0.025 | 0.433 | -0.025 | 0.010 |
| Eicosadienoic acid | -0.026 | 0.433 | -0.026 | 0.010 |
| Quinolinic acid | -0.025 | 0.433 | -0.025 | 0.010 |
| Dihydrobiopterin | -0.016 | 0.433 | -0.016 | 0.010 |
| Agavoside A | -0.018 | 0.432 | -0.018 | 0.010 |
| Linoleoyl ethanolamide | -0.015 | 0.430 | -0.015 | 0.011 |
| Pydanon | -0.018 | 0.428 | -0.018 | 0.011 |
| Stearoylcarnitine | -0.018 | 0.428 | -0.018 | 0.011 |
| 33-Dichlorobenzidine | -0.015 | 0.425 | -0.015 | 0.012 |
| Calcimycin | -0.017 | 0.424 | -0.017 | 0.012 |
| 3-Methyladipic acid | -0.015 | 0.421 | -0.015 | 0.013 |
| Adenosine monophosphate | -0.027 | 0.420 | -0.027 | 0.013 |
| Phenylglyoxylic acid | -0.018 | 0.418 | -0.018 | 0.014 |
| Sudan Brown RR | -0.021 | 0.417 | -0.021 | 0.014 |
| Thiram | -0.014 | 0.416 | -0.014 | 0.015 |
| ()-Luguine | -0.012 | 0.414 | -0.012 | 0.015 |
| Phosphoenolpyruvic acid | -0.026 | 0.414 | -0.026 | 0.015 |
| 9-Hydroxybenzoapyrene-45-oxide | -0.012 | 0.413 | -0.012 | 0.015 |
| 5-Tetrazolyl-glycine | -0.024 | 0.413 | -0.024 | 0.015 |
| (2R3R)-3-Methylglutamyl-5-semialdehyde-N6-lysine | -0.018 | 0.413 | -0.018 | 0.015 |
| Adenosine | -0.022 | 0.413 | -0.022 | 0.015 |
| PC(16:016:0) | -0.017 | 0.410 | -0.017 | 0.017 |
| Biperiden | -0.028 | 0.408 | -0.028 | 0.017 |
| Deoxycytidine | -0.021 | 0.407 | -0.021 | 0.017 |
| NA | -0.021 | 0.407 | -0.021 | 0.017 |
| 1-(4Z7Z10Z13Z16Z19Z-docosahexaenoyl)-2-(13Z16Z-docosadienoyl)-glycero-3-phosphoethanolamine | -0.024 | 0.404 | -0.024 | 0.018 |
| 2-Hydroxymethylserine | -0.017 | 0.404 | -0.017 | 0.019 |
| CI Acid Orange 20 | -0.022 | 0.400 | -0.022 | 0.020 |
| gamma-Glutamyl-beta-aminopropiononitrile | -0.019 | 0.396 | -0.019 | 0.022 |
| Isoprothiolane | -0.017 | 0.394 | -0.017 | 0.023 |
| Callinecdysone A | -0.025 | 0.391 | -0.025 | 0.024 |
| 1-tridecanoyl-2-(9Z12Z-octadecadienoyl)-glycero-3-phosphocholine | -0.025 | 0.389 | -0.025 | 0.025 |
| Indolelactic acid | -0.016 | 0.388 | -0.016 | 0.025 |
| 1-(9Z-tetradecenoyl)-2-(7Z10Z13Z16Z-docosatetraenoyl)-glycero-3-phospho-(1-myo-inositol) | -0.014 | 0.386 | -0.014 | 0.026 |
| 5-Oxoavermectin 2a aglycone | -0.022 | 0.386 | -0.022 | 0.027 |
| Carminomycin | -0.018 | 0.385 | -0.018 | 0.027 |
| 3-Methylhistamine | -0.021 | 0.379 | -0.021 | 0.030 |
| Fenthiaprop | -0.022 | 0.378 | -0.022 | 0.030 |
| 1-(9Z-tetradecenoyl)-2-(7Z10Z13Z16Z-docosatetraenoyl)-glycero-3-phosphoserine | -0.020 | 0.378 | -0.02 | 0.031 |
| Thiotepa | -0.023 | 0.378 | -0.023 | 0.031 |
| Guanosine diphosphate | -0.012 | 0.377 | -0.012 | 0.031 |
| 22-Dichloro-11-ethanediol | -0.019 | 0.376 | -0.019 | 0.032 |
| 1-(9Z-pentadecenoyl)-glycero-3-phosphocholine | -0.025 | 0.375 | -0.025 | 0.032 |
| 3-amino-octanoic acid | -0.019 | 0.373 | -0.019 | 0.034 |
| Etaconazole | -0.020 | 0.373 | -0.02 | 0.034 |
| 3-(5-Methylthio)pentylmalic acid | -0.013 | 0.371 | -0.013 | 0.035 |
| L-Alanine | -0.025 | 0.369 | -0.025 | 0.036 |
| S-Acetyldihydrolipoamide | -0.019 | 0.367 | -0.019 | 0.037 |
| 5-Methylcytosine | -0.017 | 0.365 | -0.017 | 0.039 |
| N-Acetyl-leu-leu-tyr | -0.024 | 0.364 | -0.024 | 0.039 |
| Sulochrin | -0.014 | 0.363 | -0.014 | 0.040 |
| Fumaric acid | -0.016 | 0.362 | -0.016 | 0.040 |
| CoA | -0.015 | 0.360 | -0.015 | 0.042 |
| 1-(5Z8Z11Z14Z17Z-eicosapentaenoyl)-glycero-3-phosphoethanolamine | -0.019 | 0.360 | -0.019 | 0.042 |
| Crocetin | -0.023 | 0.360 | -0.023 | 0.042 |
| Benzamidine | -0.023 | 0.358 | -0.023 | 0.044 |
| Hydnocarpic acid | -0.020 | 0.358 | -0.02 | 0.044 |
| 3-Dehydroquinate | -0.020 | 0.355 | -0.02 | 0.046 |
| 8-Hydroxyalanylclavam | -0.016 | 0.353 | -0.016 | 0.047 |
| PI(16:018:1(11Z)) | -0.016 | 0.353 | -0.016 | 0.047 |
| trans-3-Aminocyclopentane-1-carboxylic acid | -0.025 | -0.353 | -0.025 | 0.048 |
| PG(18:018:1(11Z)) | -0.023 | -0.360 | -0.023 | 0.042 |
| Deoxyadenosine | -0.024 | -0.370 | -0.024 | 0.035 |
| Trachelanthamidine | -0.027 | -0.386 | -0.027 | 0.027 |
| Vinyl toluene | -0.025 | -0.432 | -0.025 | 0.010 |
| 1-hexadecyl-2-(4Z7Z10Z13Z16Z19Z-docosahexaenoyl)-sn-glycero-3-phosphoethanolamine | -0.024 | -0.438 | -0.024 | 0.009 |
| Piperidione | -0.025 | -0.448 | -0.025 | 0.007 |
| Dopamine | -0.028 | -0.468 | -0.028 | 0.005 |
| 4-Hydroxybenzaldehyde | -0.023 | -0.475 | -0.023 | 0.004 |
| Cinobufotalin | -0.024 | -0.484 | -0.024 | 0.003 |
| Quinapril hydrochloride | -0.027 | -0.507 | -0.027 | 0.002 |
| 1-(8Z11Z14Z-eicosatrienoyl)-glycero-3-phosphoserine | -0.026 | -0.542 | -0.026 | 0.001 |
| Autumnolide | -0.027 | -0.544 | -0.027 | 0.001 |
| Robustaol A | -0.027 | -0.564 | -0.027 | 0.000 |
| Buclizine | -0.028 | -0.577 | -0.028 | 0.000 |
| Sophoranone | -0.025 | -0.585 | -0.025 | 0.000 |
| Minocycline | -0.028 | -0.587 | -0.028 | 0.000 |
| Senampeline A | -0.025 | -0.589 | -0.025 | 0.000 |
| 17-Hydroxypregnenolone sulfate | -0.028 | -0.602 | -0.028 | 0.000 |
| 3-(7-Methylthio)heptylmalic acid | -0.028 | -0.610 | -0.028 | 0.000 |
| Pteridine | -0.028 | -0.618 | -0.028 | 0.000 |
| Militarinone A | -0.027 | -0.624 | -0.027 | 0.000 |
| PA(16:018:2(9Z12Z)) | -0.026 | -0.646 | -0.026 | 0.000 |
| Naltrindole | -0.028 | -0.665 | -0.028 | 0.000 |
| 1-(9Z-octadecenoyl)-2-(9Z12Z-octadecadienoyl)-glycero-3-phosphate | -0.028 | -0.674 | -0.028 | 0.000 |
|  |  |  |  |  |

### Supplementary Table 3: Mummichog pathway enrichment for brown adipose tissue m/z values. Fold-changes and P-values relative to the 12AL control group entered. Unadjusted (P < 0.05).

| Pathway | Overlap size | Pathway size | Adjusted P-value |
| --- | --- | --- | --- |
| **40CR** |  |  |  |
| serotonin and melatonin biosynthesis | 6 | 7 | 0.025 |
| biosynthesis of serotonin and melatonin | 6 | 7 | 0.025 |
| bupropion degradation | 6 | 7 | 0.025 |
| valine degradation I | 8 | 11 | 0.028 |
| purine and pyrimidine metabolism | 11 | 17 | 0.029 |
| dolichyl-diphosphooligosaccharide biosynthesis | 4 | 4 | 0.036 |
| lipoate biosynthesis and incorporation II | 4 | 4 | 0.036 |
| adenosine nucleotides *de novo* biosynthesis | 5 | 6 | 0.038 |
| 4-aminobutyrate degradation IV | 5 | 6 | 0.038 |
| glutamate degradation IV | 6 | 8 | 0.039 |
| glycine betaine degradation | 6 | 8 | 0.039 |
| TCA cycle | 9 | 14 | 0.039 |
| folate transformations I | 8 | 12 | 0.040 |
| TCA cycle variation III (eukaryotic) | 8 | 12 | 0.040 |
| **30CR** |  |  |  |
| adenine and adenosine salvage III | 5 | 5 | 0.017 |
| aerobic respiration -- electron donors reaction list | 5 | 6 | 0.019 |
| S-adenosyl-L-methionine cycle II | 4 | 5 | 0.028 |
| methionine degradation I (to homocysteine) | 4 | 5 | 0.028 |
| lysine degradation II | 7 | 13 | 0.028 |
| glutamate degradation IV | 5 | 8 | 0.031 |
| lipoate biosynthesis and incorporation I | 3 | 3 | 0.034 |
| paraoxon degradation | 3 | 3 | 0.034 |
| threonine degradation III (to methylglyoxal) | 4 | 6 | 0.039 |
| 4-aminobutyrate degradation IV | 4 | 6 | 0.039 |
| adenosine nucleotides degradation II | 4 | 6 | 0.039 |
| purine ribonucleosides degradation to ribose-1-phosphate | 4 | 6 | 0.039 |
| TCA cycle variation III (eukaryotic) | 6 | 12 | 0.043 |
| **20CR** |  |  |  |
| catecholamine biosynthesis | 7 | 9 | 0.018 |
| adenosine nucleotides degradation II | 5 | 6 | 0.023 |
| lipoate biosynthesis and incorporation II | 4 | 4 | 0.023 |
| dopamine degradation | 6 | 9 | 0.028 |
| tyrosine degradation I | 5 | 7 | 0.030 |
| adenine and adenosine salvage III | 4 | 5 | 0.034 |
| S-adenosyl-L-methionine cycle II | 4 | 5 | 0.034 |
| methionine degradation I (to homocysteine) | 4 | 5 | 0.034 |
| L-ascorbate biosynthesis VI | 6 | 10 | 0.036 |
| lipoate biosynthesis and incorporation I | 3 | 3 | 0.039 |
| paraoxon degradation | 3 | 3 | 0.039 |
| pyrimidine ribonucleosides degradation II | 3 | 3 | 0.039 |
| lysine degradation II | 7 | 13 | 0.042 |
| **10CR** |  |  |  |
| glycine betaine degradation | 6 | 8 | 0.005 |
| TCA cycle | 7 | 14 | 0.007 |
| adenine and adenosine salvage III | 4 | 5 | 0.007 |
| Serine degradation II | 4 | 5 | 0.007 |
| glutamate degradation IV | 5 | 8 | 0.007 |
| folate transformations I | 6 | 12 | 0.009 |
| TCA cycle variation III (eukaryotic) | 6 | 12 | 0.009 |
| catecholamine biosynthesis | 5 | 9 | 0.009 |
| glycine biosynthesis I | 3 | 3 | 0.009 |
| folate polyglutamylation | 4 | 6 | 0.010 |
| 4-aminobutyrate degradation IV | 4 | 6 | 0.010 |
| lysine degradation II | 6 | 13 | 0.011 |
| L-carnitine biosynthesis | 5 | 10 | 0.012 |
| folate transformations II (plants) | 6 | 14 | 0.013 |
| glycine degradation (creatine biosynthesis) | 4 | 7 | 0.013 |
| glutamate degradation III (via 4-aminobutyrate) | 4 | 7 | 0.013 |
| 4-aminobutyrate degradation I | 4 | 7 | 0.013 |
| GDP-L-fucose biosynthesis I (from GDP-D-mannose) | 3 | 4 | 0.015 |
| aerobic respiration -- electron donor II | 3 | 4 | 0.015 |
| glutathione redox reactions II | 3 | 4 | 0.015 |
| glutathione biosynthesis | 3 | 4 | 0.015 |
| glutathione redox reactions I | 3 | 4 | 0.015 |
| glycine cleavage complex | 3 | 4 | 0.015 |
| glutathione-mediated detoxification | 3 | 4 | 0.015 |
| 5-aminoimidazole ribonucleotide biosynthesis I | 4 | 8 | 0.019 |
| D-glucuronate degradation I | 4 | 8 | 0.019 |
| methionine salvage II (mammalia) | 3 | 5 | 0.025 |
| histidine degradation III | 3 | 5 | 0.025 |
| creatine biosynthesis | 4 | 9 | 0.027 |
| histamine degradation | 4 | 9 | 0.027 |
| glycine biosynthesis III | 2 | 2 | 0.037 |
| dermatan sulfate degradation (metazoa) | 2 | 2 | 0.037 |
| bile acid biosynthesis, neutral pathway | 5 | 14 | 0.038 |
| 5-aminoimidazole ribonucleotide biosynthesis II | 3 | 6 | 0.040 |
| inosine-5'-phosphate biosynthesis II | 3 | 6 | 0.040 |
| adenosine nucleotides *de novo* biosynthesis | 3 | 6 | 0.040 |
| uracil degradation II (reductive) | 3 | 6 | 0.040 |
| serine biosynthesis | 3 | 6 | 0.040 |
| β-alanine degradation I | 3 | 6 | 0.040 |
| tryptophan degradation to 2-amino-3-carboxymuconate semialdehyde | 3 | 6 | 0.040 |
| adenosine nucleotides degradation II | 3 | 6 | 0.040 |
| purine ribonucleosides degradation to ribose-1-phosphate | 3 | 6 | 0.040 |
| tRNA charging pathway | 6 | 19 | 0.048 |
| **24AL** |  |  |  |
| TCA cycle variation III (eukaryotic) | 7 | 12 | 0.002 |
| tyrosine degradation I | 5 | 7 | 0.003 |
| TCA cycle | 7 | 14 | 0.006 |
| tRNA charging pathway | 8 | 19 | 0.012 |
| serine biosynthesis | 4 | 6 | 0.012 |
| arginine degradation VI (arginase 2 pathway) | 4 | 6 | 0.012 |
| aspartate degradation II | 4 | 6 | 0.012 |
| glutamate degradation VII | 5 | 10 | 0.021 |
| proline biosynthesis II (from arginine) | 4 | 7 | 0.023 |
| arginine degradation I (arginase pathway) | 4 | 7 | 0.023 |
| glycine degradation (creatine biosynthesis) | 4 | 7 | 0.023 |
| glutamate degradation III (via 4-aminobutyrate) | 4 | 7 | 0.023 |
| 4-aminobutyrate degradation I | 4 | 7 | 0.023 |
| aspartate biosynthesis | 3 | 4 | 0.020 |
| aerobic respiration -- electron donor II | 3 | 4 | 0.020 |
| proline degradation III | 3 | 4 | 0.020 |
| glutamine degradation II | 3 | 4 | 0.020 |
| 4-hydroxyproline degradation I | 3 | 4 | 0.020 |
| phenylalanine degradation III | 4 | 8 | 0.040 |
| glutamate degradation IV | 4 | 8 | 0.040 |
| glycine betaine degradation | 4 | 8 | 0.040 |

### Supplementary Table 4: IPA pathway enrichment for brown adipose tissue m/z values. Fold-changes and P-values relative to the 12AL control group entered. (Unadjusted P < 0.05). Top 10 pathways for each treatment group.

| Ingenuity Canonical Pathways | Ratio | Downregulated | Upregulated | P-value |
| --- | --- | --- | --- | --- |
| 40CR |  |  |  |  |
| Purine Nucleotides Degradation II (Aerobic) | 0.353 | 1/17 (6%) | 7/17 (41%) | 3.63078E-06 |
| Purine Nucleotides De Novo Biosynthesis II | 0.2 | 1/30 (3%) | 8/30 (27%) | 0.000131826 |
| Threonine Degradation II | 0.429 | 1/7 (14%) | 3/7 (43%) | 0.000691831 |
| Mitochondrial Dysfunction | 0.235 | 1/17 (6%) | 4/17 (24%) | 0.001047129 |
| Ascorbate Recycling (Cytosolic) | 0.375 | 0/8 (0%) | 3/8 (38%) | 0.001096478 |
| Phenylalanine Degradation IV (Mammalian, via Side Chain) | 0.222 | 2/18 (11%) | 6/18 (33%) | 0.001318257 |
| (S)-reticuline Biosynthesis II | 0.333 | 3/9 (33%) | 2/9 (22%) | 0.00162181 |
| NAD biosynthesis II (from tryptophan) | 0.211 | 1/19 (5%) | 7/19 (37%) | 0.00162181 |
| Tryptophan Degradation X (Mammalian, via Tryptamine) | 0.3 | 1/10 (10%) | 3/10 (30%) | 0.002238721 |
| Guanosine Nucleotides Degradation III | 0.3 | 1/10 (10%) | 3/10 (30%) | 0.002238721 |
| Bile Acid Biosynthesis, Neutral Pathway | 0.139 | 0/36 (0%) | 6/36 (17%) | 0.002884032 |
| Serotonin and Melatonin Biosynthesis | 0.273 | 1/11 (9%) | 4/11 (36%) | 0.003019952 |
| Catecholamine Biosynthesis | 0.273 | 3/11 (27%) | 2/11 (18%) | 0.003019952 |
| Adenosine Nucleotides Degradation II | 0.273 | 0/11 (0%) | 4/11 (36%) | 0.003019952 |
| Arsenate Detoxification I (Glutaredoxin) | 0.25 | 1/12 (8%) | 3/12 (25%) | 0.003981072 |
| Oxidative Ethanol Degradation III | 0.25 | 0/12 (0%) | 4/12 (33%) | 0.003981072 |
| Glutathione Redox Reactions II | 0.5 | 0/4 (0%) | 2/4 (50%) | 0.004570882 |
| Adenine and Adenosine Salvage VI | 0.5 | 0/4 (0%) | 2/4 (50%) | 0.004570882 |
| Dopamine Degradation | 0.214 | 2/14 (14%) | 3/14 (21%) | 0.006309573 |
| 4-hydroxybenzoate Biosynthesis | 0.214 | 2/14 (14%) | 4/14 (29%) | 0.006309573 |
| tRNA Charging | 0.116 | 9/43 (21%) | 7/43 (16%) | 0.006309573 |
| Spermine Biosynthesis | 0.4 | 0/5 (0%) | 3/5 (60%) | 0.007585776 |
| Spermidine Biosynthesis I | 0.4 | 1/5 (20%) | 3/5 (60%) | 0.007585776 |
| Taurine Biosynthesis | 0.333 | 1/6 (17%) | 3/6 (50%) | 0.011220185 |
| Antioxidant Action of Vitamin C | 0.333 | 0/6 (0%) | 2/6 (33%) | 0.011220185 |
| Glycine Biosynthesis II | 0.333 | 0/6 (0%) | 2/6 (33%) | 0.011220185 |
| Sumoylation Pathway | 0.333 | 0/6 (0%) | 2/6 (33%) | 0.011220185 |
| AMPK Signaling | 0.176 | 0/17 (0%) | 5/17 (29%) | 0.011220185 |
| Salvage Pathways of Pyrimidine Ribonucleotides | 0.176 | 0/17 (0%) | 6/17 (35%) | 0.011220185 |
| Folate Transformations I | 0.158 | 1/19 (5%) | 4/19 (21%) | 0.015135612 |
| Uracil Degradation II (Reductive) | 0.286 | 0/7 (0%) | 2/7 (29%) | 0.015135612 |
| Glutathione Redox Reactions I | 0.286 | 0/7 (0%) | 2/7 (29%) | 0.015135612 |
| tRNA Splicing | 0.286 | 0/7 (0%) | 3/7 (43%) | 0.015135612 |
| Guanine and Guanosine Salvage I | 0.286 | 1/7 (14%) | 2/7 (29%) | 0.015135612 |
| Phenylethylamine Degradation I | 0.286 | 1/7 (14%) | 1/7 (14%) | 0.015135612 |
| NAD Phosphorylation and Dephosphorylation | 0.286 | 0/7 (0%) | 2/7 (29%) | 0.015135612 |
| Glycine Cleavage Complex | 0.286 | 0/7 (0%) | 2/7 (29%) | 0.015135612 |
| Methylglyoxal Degradation III | 0.286 | 0/7 (0%) | 2/7 (29%) | 0.015135612 |
| Glycine Degradation (Creatine Biosynthesis) | 0.286 | 3/7 (43%) | 4/7 (57%) | 0.015135612 |
| Adenine and Adenosine Salvage I | 0.286 | 0/7 (0%) | 2/7 (29%) | 0.015135612 |
| Phenylalanine Degradation I (Aerobic) | 0.286 | 2/7 (29%) | 2/7 (29%) | 0.015135612 |
| Vitamin-C Transport | 0.286 | 0/7 (0%) | 2/7 (29%) | 0.015135612 |
| Lipoate Biosynthesis and Incorporation II | 0.25 | 1/8 (13%) | 2/8 (25%) | 0.019952623 |
| D-glucuronate Degradation I | 0.25 | 0/8 (0%) | 2/8 (25%) | 0.019952623 |
| Methionine Degradation I (to Homocysteine) | 0.25 | 2/8 (25%) | 2/8 (25%) | 0.019952623 |
| Cardiac β-adrenergic Signaling | 0.25 | 0/8 (0%) | 3/8 (38%) | 0.019952623 |
| Phototransduction Pathway | 0.25 | 0/8 (0%) | 3/8 (38%) | 0.019952623 |
| Gluconeogenesis I | 0.143 | 2/21 (10%) | 4/21 (19%) | 0.019952623 |
| Diphthamide Biosynthesis | 0.222 | 1/9 (11%) | 2/9 (22%) | 0.025118864 |
| NAD Salvage Pathway II | 0.222 | 0/9 (0%) | 2/9 (22%) | 0.025118864 |
| Urate Biosynthesis/Inosine 5'-phosphate Degradation | 0.222 | 0/9 (0%) | 2/9 (22%) | 0.025118864 |
| dTMP De Novo Biosynthesis | 0.222 | 0/9 (0%) | 3/9 (33%) | 0.025118864 |
| Molybdenum Cofactor Biosynthesis | 0.222 | 1/9 (11%) | 3/9 (33%) | 0.025118864 |
| Relaxin Signaling | 0.2 | 1/10 (10%) | 2/10 (20%) | 0.030902954 |
| L-carnitine Biosynthesis | 0.2 | 0/10 (0%) | 3/10 (30%) | 0.030902954 |
| NAD Biosynthesis from 2-amino-3-carboxymuconate Semialdehyde | 0.2 | 1/10 (10%) | 4/10 (40%) | 0.030902954 |
| Sphingosine and Sphingosine-1-phosphate Metabolism | 0.2 | 0/10 (0%) | 3/10 (30%) | 0.030902954 |
| Phosphatidylethanolamine Biosynthesis II | 0.2 | 0/10 (0%) | 3/10 (30%) | 0.030902954 |
| Nicotine Degradation II | 0.2 | 1/10 (10%) | 3/10 (30%) | 0.030902954 |
| Adenine and Adenosine Salvage III | 0.2 | 0/10 (0%) | 3/10 (30%) | 0.030902954 |
| Tryptophan Degradation to 2-amino-3-carboxymuconate Semialdehyde | 0.2 | 0/10 (0%) | 3/10 (30%) | 0.030902954 |
| Glucocorticoid Biosynthesis | 0.2 | 0/10 (0%) | 2/10 (20%) | 0.030902954 |
| Ethanol Degradation II | 0.2 | 0/10 (0%) | 3/10 (30%) | 0.030902954 |
| Serotonin Receptor Signaling | 0.2 | 0/10 (0%) | 3/10 (30%) | 0.030902954 |
| Tryptophan Degradation III (Eukaryotic) | 0.12 | 0/25 (0%) | 7/25 (28%) | 0.032359366 |
| L-DOPA Degradation | 0.182 | 1/11 (9%) | 3/11 (27%) | 0.037153523 |
| Citrulline-Nitric Oxide Cycle | 0.182 | 2/11 (18%) | 3/11 (27%) | 0.037153523 |
| Mineralocorticoid Biosynthesis | 0.182 | 0/11 (0%) | 2/11 (18%) | 0.037153523 |
| Ethanol Degradation IV | 0.182 | 0/11 (0%) | 3/11 (27%) | 0.037153523 |
| Androgen Biosynthesis | 0.167 | 0/12 (0%) | 2/12 (17%) | 0.043651583 |
| Superpathway of Serine and Glycine Biosynthesis I | 0.167 | 0/12 (0%) | 4/12 (33%) | 0.043651583 |
| The Visual Cycle | 0.167 | 0/12 (0%) | 3/12 (25%) | 0.043651583 |
| Lysine Degradation II | 0.167 | 0/12 (0%) | 5/12 (42%) | 0.043651583 |
| Purine Ribonucleosides Degradation to Ribose-1-phosphate | 0.167 | 1/12 (8%) | 3/12 (25%) | 0.043651583 |
| Salvage Pathways of Pyrimidine Deoxyribonucleotides | 0.167 | 1/12 (8%) | 2/12 (17%) | 0.043651583 |
| 30CR |  |  |  |  |
| Purine Nucleotides Degradation II (Aerobic) | 0.176 | 0/17 (0%) | 8/17 (47%) | 0.000812831 |
| Guanine and Guanosine Salvage I | 0.286 | 0/7 (0%) | 3/7 (43%) | 0.002630268 |
| Glycine Degradation (Creatine Biosynthesis) | 0.286 | 2/7 (29%) | 5/7 (71%) | 0.002630268 |
| Threonine Degradation II | 0.286 | 2/7 (29%) | 2/7 (29%) | 0.002630268 |
| Phenylalanine Degradation I (Aerobic) | 0.286 | 1/7 (14%) | 3/7 (43%) | 0.002630268 |
| (S)-reticuline Biosynthesis II | 0.222 | 4/9 (44%) | 1/9 (11%) | 0.004466836 |
| Purine Nucleotides De Novo Biosynthesis II | 0.1 | 3/30 (10%) | 6/30 (20%) | 0.004466836 |
| Guanosine Nucleotides Degradation III | 0.2 | 0/10 (0%) | 4/10 (40%) | 0.005495409 |
| Catecholamine Biosynthesis | 0.182 | 3/11 (27%) | 2/11 (18%) | 0.006606934 |
| Arsenate Detoxification I (Glutaredoxin) | 0.167 | 1/12 (8%) | 3/12 (25%) | 0.007943282 |
| Salvage Pathways of Pyrimidine Deoxyribonucleotides | 0.167 | 1/12 (8%) | 2/12 (17%) | 0.007943282 |
| Dopamine Receptor Signaling | 0.143 | 2/14 (14%) | 1/14 (7%) | 0.010715193 |
| tRNA Charging | 0.0698 | 6/43 (14%) | 10/43 (23%) | 0.012302688 |
| Salvage Pathways of Pyrimidine Ribonucleotides | 0.118 | 1/17 (6%) | 5/17 (29%) | 0.015848932 |
| Phenylalanine Degradation IV (Mammalian, via Side Chain) | 0.111 | 4/18 (22%) | 4/18 (22%) | 0.017782794 |
| Bupropion Degradation | 0.5 | 0/2 (0%) | 1/2 (50%) | 0.023442288 |
| Wnt/Ca+ pathway | 0.333 | 0/3 (0%) | 1/3 (33%) | 0.034673685 |
| L-dopachrome Biosynthesis | 0.25 | 1/4 (25%) | 0/4 (0%) | 0.045708819 |
| Glycine Biosynthesis III | 0.25 | 0/4 (0%) | 2/4 (50%) | 0.045708819 |
| Glycine Biosynthesis I | 0.25 | 0/4 (0%) | 2/4 (50%) | 0.045708819 |
| Tyrosine Biosynthesis IV | 0.25 | 1/4 (25%) | 1/4 (25%) | 0.045708819 |
| 4-hydroxyphenylpyruvate Biosynthesis | 0.25 | 3/4 (75%) | 0/4 (0%) | 0.045708819 |
| 20CR |  |  |  |  |
| (S)-reticuline Biosynthesis II | 0.444 | 3/9 (33%) | 2/9 (22%) | 7.24436E-06 |
| 4-hydroxyphenylpyruvate Biosynthesis | 0.75 | 2/4 (50%) | 1/4 (25%) | 1.65959E-05 |
| Catecholamine Biosynthesis | 0.364 | 3/11 (27%) | 2/11 (18%) | 1.86209E-05 |
| 4-hydroxybenzoate Biosynthesis | 0.286 | 2/14 (14%) | 4/14 (29%) | 5.49541E-05 |
| Purine Nucleotides Degradation II (Aerobic) | 0.235 | 0/17 (0%) | 8/17 (47%) | 0.000125893 |
| Tyrosine Degradation I | 0.333 | 4/9 (44%) | 1/9 (11%) | 0.000331131 |
| Cellular Effects of Sildenafil (Viagra) | 0.231 | 1/13 (8%) | 2/13 (15%) | 0.001071519 |
| Gap Junction Signaling | 0.231 | 1/13 (8%) | 3/13 (23%) | 0.001071519 |
| Purine Nucleotides De Novo Biosynthesis II | 0.133 | 1/30 (3%) | 8/30 (27%) | 0.001258925 |
| Dopamine Receptor Signaling | 0.214 | 2/14 (14%) | 1/14 (7%) | 0.001348963 |
| Aspartate Biosynthesis | 0.5 | 1/4 (25%) | 1/4 (25%) | 0.001584893 |
| nNOS Signaling in Neurons | 0.4 | 1/5 (20%) | 2/5 (40%) | 0.002630268 |
| Arginine Biosynthesis IV | 0.167 | 2/18 (11%) | 5/18 (28%) | 0.002884032 |
| Glutamate Degradation II | 0.333 | 2/6 (33%) | 1/6 (17%) | 0.003890451 |
| tRNA Charging | 0.093 | 6/43 (14%) | 10/43 (23%) | 0.004897788 |
| Glycine Degradation (Creatine Biosynthesis) | 0.286 | 3/7 (43%) | 4/7 (57%) | 0.005370318 |
| Asparagine Biosynthesis I | 0.286 | 0/7 (0%) | 3/7 (43%) | 0.005370318 |
| Phenylalanine Degradation I (Aerobic) | 0.286 | 1/7 (14%) | 3/7 (43%) | 0.005370318 |
| Aspartate Degradation II | 0.286 | 1/7 (14%) | 3/7 (43%) | 0.005370318 |
| Superpathway of Citrulline Metabolism | 0.125 | 3/24 (13%) | 6/24 (25%) | 0.00676083 |
| Lipoate Biosynthesis and Incorporation II | 0.25 | 0/8 (0%) | 3/8 (38%) | 0.007079458 |
| Cardiac β-adrenergic Signaling | 0.25 | 0/8 (0%) | 3/8 (38%) | 0.007079458 |
| L-glutamine Biosynthesis II (tRNA-dependent) | 0.222 | 0/9 (0%) | 3/9 (33%) | 0.009120108 |
| Arginine Degradation I (Arginase Pathway) | 0.222 | 1/9 (11%) | 3/9 (33%) | 0.009120108 |
| Relaxin Signaling | 0.2 | 1/10 (10%) | 2/10 (20%) | 0.011220185 |
| NAD Biosynthesis from 2-amino-3-carboxymuconate Semialdehyde | 0.2 | 0/10 (0%) | 5/10 (50%) | 0.011220185 |
| Arginine Degradation VI (Arginase 2 Pathway) | 0.2 | 2/10 (20%) | 3/10 (30%) | 0.011220185 |
| Adenine and Adenosine Salvage III | 0.2 | 0/10 (0%) | 3/10 (30%) | 0.011220185 |
| L-DOPA Degradation | 0.182 | 1/11 (9%) | 3/11 (27%) | 0.013489629 |
| Adenosine Nucleotides Degradation II | 0.182 | 0/11 (0%) | 4/11 (36%) | 0.013489629 |
| Citrulline-Nitric Oxide Cycle | 0.182 | 2/11 (18%) | 3/11 (27%) | 0.013489629 |
| Arsenate Detoxification I (Glutaredoxin) | 0.167 | 1/12 (8%) | 3/12 (25%) | 0.016218101 |
| Salvage Pathways of Pyrimidine Deoxyribonucleotides | 0.167 | 1/12 (8%) | 2/12 (17%) | 0.016218101 |
| Proline Biosynthesis II (from Arginine) | 0.154 | 2/13 (15%) | 4/13 (31%) | 0.019054607 |
| γ-glutamyl Cycle | 0.154 | 1/13 (8%) | 3/13 (23%) | 0.019054607 |
| Nitric Oxide Signaling in the Cardiovascular System | 0.154 | 1/13 (8%) | 3/13 (23%) | 0.019054607 |
| Dopamine-DARPP32 Feedback in cAMP Signaling | 0.143 | 1/14 (7%) | 2/14 (14%) | 0.021877616 |
| Dopamine Degradation | 0.143 | 2/14 (14%) | 3/14 (21%) | 0.021877616 |
| Urea Cycle | 0.143 | 2/14 (14%) | 3/14 (21%) | 0.021877616 |
| Histamine Degradation | 0.143 | 1/14 (7%) | 3/14 (21%) | 0.021877616 |
| Salvage Pathways of Pyrimidine Ribonucleotides | 0.118 | 0/17 (0%) | 6/17 (35%) | 0.031622777 |
| Noradrenaline and Adrenaline Degradation | 0.118 | 1/17 (6%) | 4/17 (24%) | 0.031622777 |
| Circadian Rhythm Signaling | 0.5 | 0/2 (0%) | 1/2 (50%) | 0.033113112 |
| Glutamate Removal from Folates | 0.5 | 0/2 (0%) | 1/2 (50%) | 0.033113112 |
| Glutamate Dependent Acid Resistance | 0.5 | 0/2 (0%) | 1/2 (50%) | 0.033113112 |
| Citrulline Biosynthesis | 0.111 | 2/18 (11%) | 5/18 (28%) | 0.035481339 |
| Phenylalanine Degradation IV (Mammalian, via Side Chain) | 0.111 | 1/18 (6%) | 7/18 (39%) | 0.035481339 |
| NAD biosynthesis II (from tryptophan) | 0.105 | 0/19 (0%) | 8/19 (42%) | 0.038904514 |
| Gluconeogenesis I | 0.0952 | 1/21 (5%) | 5/21 (24%) | 0.046773514 |
| 10CR |  |  |  |  |
| Glycine Degradation (Creatine Biosynthesis) | 0.429 | 2/7 (29%) | 5/7 (71%) | 5.49541E-05 |
| Glycine Biosynthesis I | 0.5 | 0/4 (0%) | 2/4 (50%) | 0.000870964 |
| Purine Nucleotides Degradation II (Aerobic) | 0.176 | 0/17 (0%) | 8/17 (47%) | 0.000977237 |
| dTMP De Novo Biosynthesis | 0.222 | 0/9 (0%) | 3/9 (33%) | 0.005011872 |
| Purine Nucleotides De Novo Biosynthesis II | 0.1 | 1/30 (3%) | 8/30 (27%) | 0.005248075 |
| Adenine and Adenosine Salvage III | 0.2 | 0/10 (0%) | 3/10 (30%) | 0.00616595 |
| Catecholamine Biosynthesis | 0.182 | 3/11 (27%) | 2/11 (18%) | 0.007585776 |
| Superpathway of Serine and Glycine Biosynthesis I | 0.167 | 0/12 (0%) | 4/12 (33%) | 0.008912509 |
| γ-glutamyl Cycle | 0.154 | 1/13 (8%) | 3/13 (23%) | 0.010471285 |
| Glycine Betaine Degradation | 0.154 | 1/13 (8%) | 2/13 (15%) | 0.010471285 |
| Folate Polyglutamylation | 0.154 | 0/13 (0%) | 3/13 (23%) | 0.010471285 |
| Cysteine Biosynthesis III (mammalia) | 0.154 | 3/13 (23%) | 3/13 (23%) | 0.010471285 |
| Noradrenaline and Adrenaline Degradation | 0.118 | 1/17 (6%) | 4/17 (24%) | 0.017782794 |
| Folate Transformations I | 0.105 | 1/19 (5%) | 4/19 (21%) | 0.021877616 |
| Bupropion Degradation | 0.5 | 0/2 (0%) | 1/2 (50%) | 0.024547089 |
| Putrescine Biosynthesis III | 0.5 | 0/2 (0%) | 2/2 (100%) | 0.024547089 |
| L-serine Degradation | 0.333 | 0/3 (0%) | 1/3 (33%) | 0.037153523 |
| Wnt/Ca+ pathway | 0.333 | 0/3 (0%) | 1/3 (33%) | 0.037153523 |
| Glycine Biosynthesis III | 0.25 | 0/4 (0%) | 2/4 (50%) | 0.048977882 |
| Phosphatidylethanolamine Biosynthesis III | 0.25 | 0/4 (0%) | 1/4 (25%) | 0.048977882 |
| 24AL |  |  |  |  |
| Glycine Degradation (Creatine Biosynthesis) | 0.429 | 1/7 (14%) | 6/7 (86%) | 6.60693E-05 |
| Cysteine Biosynthesis III (mammalia) | 0.231 | 2/13 (15%) | 4/13 (31%) | 0.000512861 |
| 4-hydroxyphenylpyruvate Biosynthesis | 0.5 | 2/4 (50%) | 1/4 (25%) | 0.000977237 |
| Spermine Biosynthesis | 0.4 | 0/5 (0%) | 3/5 (60%) | 0.00162181 |
| Spermidine Biosynthesis I | 0.4 | 0/5 (0%) | 4/5 (80%) | 0.00162181 |
| tRNA Charging | 0.093 | 7/43 (16%) | 9/43 (21%) | 0.001905461 |
| Methionine Degradation I (to Homocysteine) | 0.25 | 1/8 (13%) | 3/8 (38%) | 0.004365158 |
| Diphthamide Biosynthesis | 0.222 | 0/9 (0%) | 3/9 (33%) | 0.005623413 |
| (S)-reticuline Biosynthesis II | 0.222 | 1/9 (11%) | 4/9 (44%) | 0.005623413 |
| Tyrosine Degradation I | 0.222 | 3/9 (33%) | 2/9 (22%) | 0.005623413 |
| Catecholamine Biosynthesis | 0.182 | 1/11 (9%) | 4/11 (36%) | 0.00851138 |
| Superpathway of Methionine Degradation | 0.0882 | 3/34 (9%) | 6/34 (18%) | 0.008912509 |
| Dopamine Degradation | 0.143 | 1/14 (7%) | 4/14 (29%) | 0.013489629 |
| 4-hydroxybenzoate Biosynthesis | 0.143 | 3/14 (21%) | 3/14 (21%) | 0.013489629 |
| Putrescine Biosynthesis III | 0.5 | 0/2 (0%) | 2/2 (100%) | 0.02630268 |
| Acyl Carrier Protein Metabolism | 0.333 | 0/3 (0%) | 2/3 (67%) | 0.038904514 |
| L-serine Degradation | 0.333 | 0/3 (0%) | 1/3 (33%) | 0.038904514 |

### Supplementary Table 5: Correlation of metabolites with physical parameters, CR treatment level, body temperature over the final two weeks (Tb), food anticipatory activity over the final two weeks (FAA) and physical activity over the final two weeks (PA). Benjamini-Hochberg adjusted p-value.

| Metabolite | CR level | | Tb | | FAA | | PA | |
| --- | --- | --- | --- | --- | --- | --- | --- | --- |
|  | R | P-value | R | P-value | R | P-value | R | P-value |
| Serotonin | 0.415 | 0.070 | -0.454 | 0.040 | 0.324 | 0.163 | 0.234 | 0.664 |
| Dopamine | -0.365 | 0.031 | 0.138 | 0.348 | -0.237 | 0.080 | -0.162 | 0.596 |
| L-Serine | -0.109 | 0.935 | 0.167 | 0.857 | -0.062 | 0.962 | -0.084 | 0.945 |
| Oxidized glutathione | 0.222 | 0.048 | -0.265 | 0.228 | 0.368 | 0.158 | 0.713 | 0.229 |
| 3-Methylhistamine | 0.470 | 0.006 | -0.351 | 0.050 | 0.536 | 0.014 | 0.141 | 0.832 |
| Uracil | 0.478 | 0.015 | -0.431 | 0.081 | 0.449 | 0.049 | 0.109 | 0.801 |
| Adenosine | -0.078 | 0.791 | 0.055 | 0.926 | -0.210 | 0.641 | -0.085 | 0.868 |
| 2-Aminoadenosine | 0.867 | 0.000 | -0.816 | 0.000 | 0.864 | 0.000 | 0.239 | 0.533 |
| Guanosine | 0.544 | 0.003 | -0.454 | 0.080 | 0.623 | 0.009 | 0.400 | 0.330 |
| Cytosine | 0.145 | 0.675 | -0.119 | 0.708 | 0.317 | 0.363 | 0.587 | 0.222 |
| AMP | 0.292 | 0.123 | -0.298 | 0.228 | 0.299 | 0.140 | 0.199 | 0.669 |
| NADPH | 0.359 | 0.191 | -0.373 | 0.158 | 0.400 | 0.086 | 0.400 | 0.419 |
| NAD | 0.399 | 0.003 | -0.395 | 0.142 | 0.336 | 0.121 | 0.254 | 0.469 |
| S-Adenosyl-L-methionine | 0.509 | 0.046 | -0.474 | 0.092 | 0.525 | 0.068 | 0.342 | 0.619 |

### Supplementary Table 6: Pathways potentially associated with longevity in brown adipose tissue (BAT). Metabolites were correlated with the level of CR, which has been shown to have a linear relationship with increasing lifespan. Correlation coefficients and p-values were entered into mummichog to indicate pathways that may be associated with increasing lifespan in BAT.

| Pathway | Overlap size | Pathway size | Adjusted P-value |
| --- | --- | --- | --- |
| adenine and adenosine salvage III | 5 | 5 | 0.015 |
| glutamate degradation IV | 6 | 8 | 0.017 |
| 4-aminobutyrate degradation IV | 5 | 6 | 0.018 |
| purine ribonucleosides degradation to ribose-1-phosphate | 5 | 6 | 0.018 |
| glutamate degradation III (via 4-aminobutyrate) | 5 | 7 | 0.022 |
| 4-aminobutyrate degradation I | 5 | 7 | 0.022 |
| bupropion degradation | 5 | 7 | 0.022 |
| tryptophan degradation I (via anthranilate) | 3 | 3 | 0.033 |
| TCA cycle | 7 | 14 | 0.035 |
| aerobic respiration -- electron donors reaction list | 4 | 6 | 0.039 |
| tryptophan degradation to 2-amino-3-carboxymuconate semialdehyde | 4 | 6 | 0.039 |
| adenosine nucleotides degradation II | 4 | 6 | 0.039 |
| dopamine degradation | 5 | 9 | 0.043 |
| TCA cycle variation III (eukaryotic) | 6 | 12 | 0.044 |
